# The influence of physiological and pathological perturbations on blood-brain barrier function

**DOI:** 10.3389/fnins.2023.1289894

**Published:** 2023-10-23

**Authors:** Nan Zhao, Tracy D. Chung, Zhaobin Guo, John J. Jamieson, Lily Liang, Raleigh M. Linville, Alex F. Pessell, Linus Wang, Peter C. Searson

**Affiliations:** ^1^Institute for Nanobiotechnology, Johns Hopkins University, Baltimore, MD, United States; ^2^Department of Biomedical Engineering, Johns Hopkins University, Baltimore, MD, United States; ^3^Department of Chemical and Biomolecular Engineering, Johns Hopkins University, Baltimore, MD, United States; ^4^Department of Materials Science and Engineering, Johns Hopkins University, Baltimore, MD, United States

**Keywords:** blood-brain barrier, perturbations, dysfunction, brain health, neurovascular unit, brain pathologies

## Abstract

The blood-brain barrier (BBB) is located at the interface between the vascular system and the brain parenchyma, and is responsible for communication with systemic circulation and peripheral tissues. During life, the BBB can be subjected to a wide range of perturbations or stresses that may be endogenous or exogenous, pathological or therapeutic, or intended or unintended. The risk factors for many diseases of the brain are multifactorial and involve perturbations that may occur simultaneously (e.g., two-hit model for Alzheimer’s disease) and result in different outcomes. Therefore, it is important to understand the influence of individual perturbations on BBB function in isolation. Here we review the effects of eight perturbations: mechanical forces, temperature, electromagnetic radiation, hypoxia, endogenous factors, exogenous factors, chemical factors, and pathogens. While some perturbations may result in acute or chronic BBB disruption, many are also exploited for diagnostic or therapeutic purposes. The resultant outcome on BBB function depends on the dose (or magnitude) and duration of the perturbation. Homeostasis may be restored by self-repair, for example, via processes such as proliferation of affected cells or angiogenesis to create new vasculature. Transient or sustained BBB dysfunction may result in acute or pathological symptoms, for example, microhemorrhages or hypoperfusion. In more extreme cases, perturbations may lead to cytotoxicity and cell death, for example, through exposure to cytotoxic plaques.

## 1. Introduction

**Figure 1:**
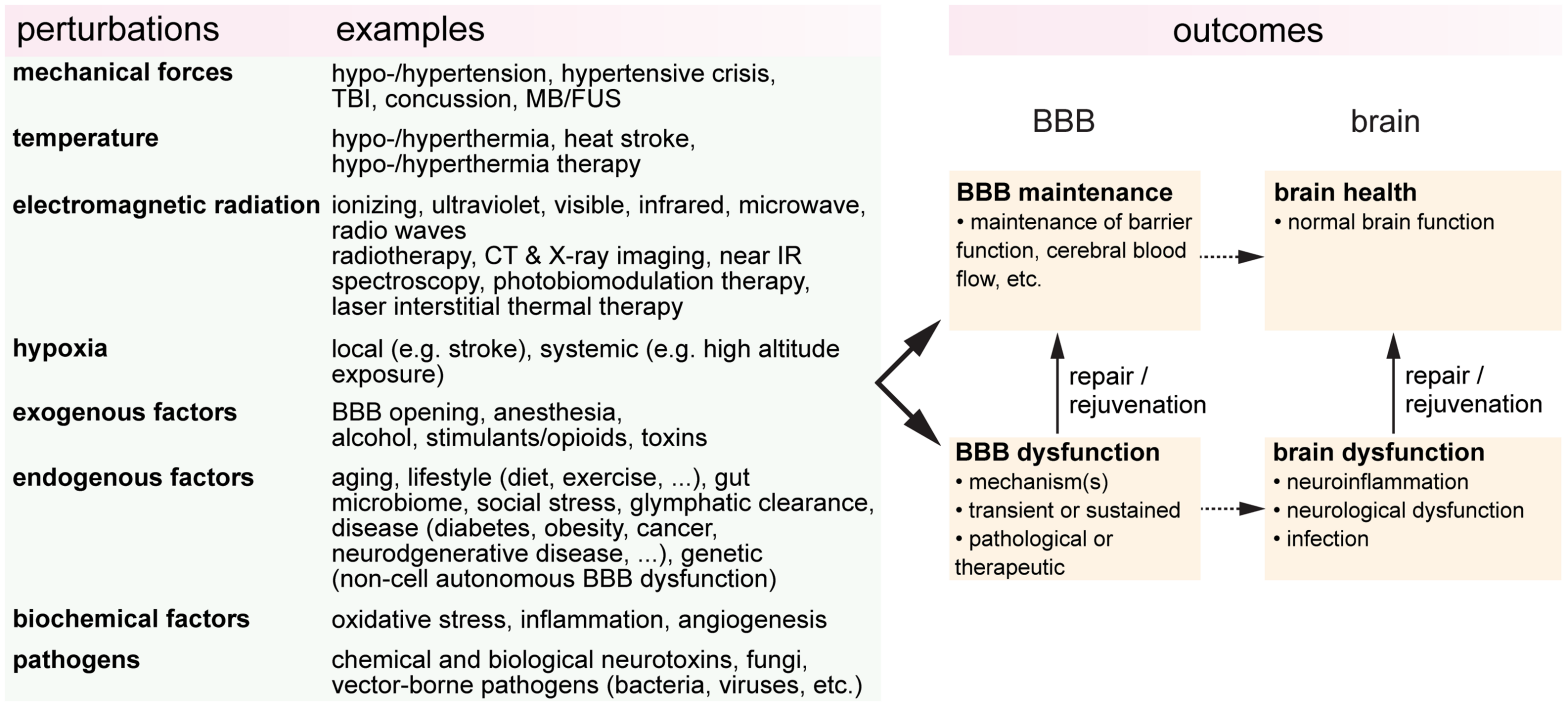
A wide range of perturbations can modulate blood-brain barrier (BBB function). These perturbations may be transient or sustained, endogenous or exogenous, pathological or therapeutic, or intended or unintended. TBI, traumatic brain injury; FUS, focused ultrasound; CT, computerized tomography; IR, infrared; BBBO, blood-brain barrier opening.

It is increasingly evident that blood-brain barrier (BBB) health is important in promoting brain health, and that BBB dysfunction is a biomarker for various brain pathologies. BBB dysfunction is usually considered in the context of disease, however, during life, the BBB can be subjected to a wide range of perturbations or stresses (Figure 1). These perturbations may be endogenous or exogenous, pathological or therapeutic, or intended or unintended. The resultant outcomes are dependent on the dose and duration, and the combinatorial or cumulative effects of multiple perturbations. To establish the relationships between BBB health and brain health, it is essential to understand how different perturbations influence outcomes. Here we review the effects of eight perturbations on the BBB: mechanical forces, temperature, electromagnetic radiation, hypoxia, endogenous factors, exogenous factors, biochemical factors, and pathogens.

### 1.1. The blood-brain barrier

**Figure 2:**
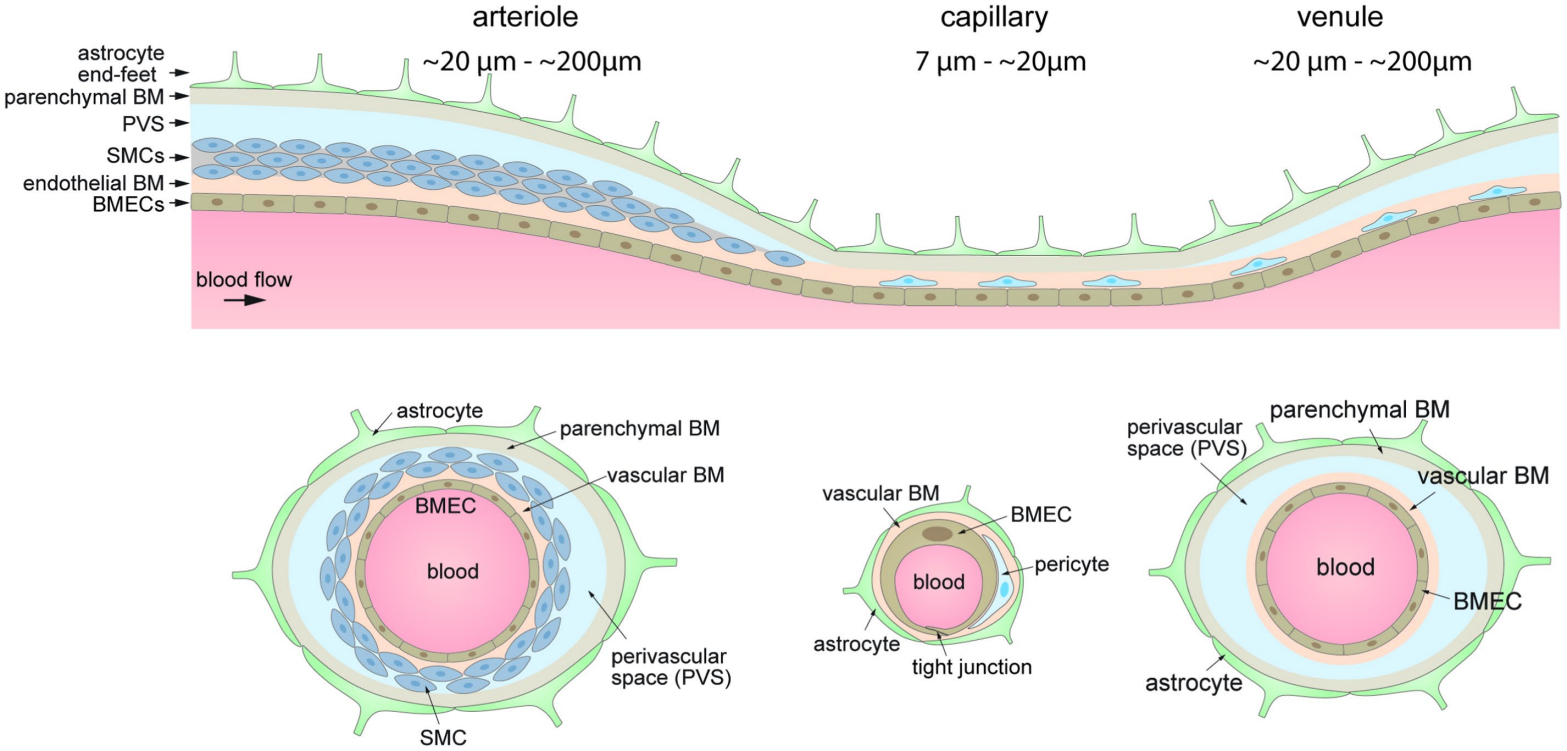
Organization of the cells of the neurovascular unit along the arterio-venous axis.

The neurovascular unit (NVU) is the functional building block of the blood-brain barrier (BBB) and includes brain microvascular endothelial cells (BMECs), supporting cells (e.g., pericytes and astrocytes), and the endothelial and parenchymal basement membranes (BMs) (Figure 2). Deletion/knock-out and functional studies have been used to infer the role of specific genes on BBB function, providing an expanding knowledge base of NVU components along the arterio-venous axis (Sweeney et al., 2019). The highly specialized BMECs provide the physical barrier between circulation and the brain. Recent transcriptomic atlases for mouse and human brain have highlighted differences in gene expression of cells in the NVU along the arterio-venous axis (Vanlandewijck et al., 2018; Garcia et al., 2022; Jeong et al., 2022; Yang A. C. et al., 2022; Crouch et al., 2023; Sun et al., 2023). For details of BBB structure readers are referred to these and other reviews.

The formation of tight junctions between BMECs effectively blocks paracellular transport of larger molecules and hence passage into and out of the brain relies on a variety of transport systems (Table 1). Passive transport is limited to small lipophilic molecules and gasses (O_2_ and CO_2_). Carrier mediated transport utilizes solute carriers (SLCs) for transport of nutrients and other essential molecules to the brain. Efflux pumps (e.g., ATP binding cassette (ABCs)) return many of the small lipophilic molecules capable of passive diffusion across the luminal membrane back into circulation (Loscher and Potschka, 2005). Vesicular trafficking via receptor mediated transport or adsorption mediated transport regulates transport of proteins and peptides into and out of the brain. Receptor-mediated transport involves ligand binding, most commonly via clathrin-mediated endocytosis. Adsorption mediated transport is initiated by electrostatic interactions between a ligand and the glycocalyx and usually involves caveolae-mediated transcytosis. Non-specific vesicular trafficking is generally suppressed in the healthy brain. Ion transporters are also important for regulating electrolyte balance. As a result of the high energy demand for maintenance of these systems, BMECs have more mitochondria compared ECs in other tissues (Oldendorf et al., 1977). For details of BBB structure in health and disease, readers are referred to recent reviews (Wong et al., 2013; Sweeney et al., 2018, 2019; Banks et al., 2021; Schaeffer and Iadecola, 2021).

**Table 1:** Blood-brain barrier (BBB) components and examples of dysfunction.

BBB function	Molecular constituents	Dysfunction	Examples
Paracellular transport	Tight junctions (TJs) expressed by BMECs (e.g., claudin-5, occludin, ZO-1, …)	↓ TJs – ↑ paracellular transport Cell loss – ↑ paracellular transport • Mechanical disruption – ↑ paracellular transport	Leaky brain: microbleeds or microhemorrhages (AD, CAA, stroke, TBI, healthy aging)
Passive transport	Small (< 500 Da) lipophilic molecules	See ATP-binding cassette efflux pumps (ABCs)	
Carrier mediated transport: solute carrier transporters (SLCs)	Energy transport (e.g., GLUT-1), amino acid transport (e.g., LAT-1), organic anion/cation transport (e.g., OATP1A2), nucleotides	↓ GLUT-1 – ↓ nutrient transport ↓ LAT-1 – ↓ protein & nucleotide synthesis, metabolism	Changes in respiration of cells in NVU
ATP-binding cassette efflux pumps (ABCs)	P-glycoprotein (P-gp, ABCB1), BCRP (ABCG2), MRP1 (ABCC1)	↓ P-gp – ↑ passive transport of substrates, ↓ clearance of Aβ	
Vesicular trafficking I receptor-mediated transport	Transferrin receptor (TfR), insulin receptor (IR), leptin receptor (LEP-R), low density lipoprotein receptor 1&2 (LRP1/2), receptor for advanced glycation end products (RAGE)	↓ LRP1 – ↓ clearance of Aβ and APOE 2/3	
Vesicular trafficking II adsorption mediated transport	Histone, albumin	↓ MFSD2A – ↑ caveola-mediated vesicular transport	Shift to non-specific transport in aging
Ion transporters	Sodium pumps, calcium transporters, and potassium channels	Changes in ionic homeostasis	
Other processes involving BMECs	Wound healing response, activation	Activation – ↑ adhesion molecules (e.g., ICAM-1)	
Supporting cells	Loss or degeneration of SMCs, PCs; detachment of astrocytic end-feet	↓ Signaling between BMECs and supporting cells	AD
Basement membrane	Endothelial, parenchymal (collagen IV, laminin, perlecan, agrin, nidogen)	↑ Thickness in aging	Aging, AD

BMECs, brain microvascular endothelial cells; SMCs, smooth muscle cells; PCs, pericytes; TJs, tight junctions; AD, Alzheimer’s disease; CAA, cerebral amyloid angiopathy; TBI, traumatic brain injury; ZO-1, zona occludens 1; GLUT-1, glucose transporter 1; LAT-1, large neutral amino acids transporter 1; BCRP, breast cancer resistance protein; ICAM-1, intercellular adhesion molecule 1; MFSD2A, major facilitator superfamily domain-containing protein 2; APOE, apolipoprotein E.

### 1.2. BBB dysfunction

BBB dysfunction can include any gain or loss of function that has the potential to induce pathological changes in the brain. In general, BBB dysfunction can be broadly classified as: (1) processes that directly or indirectly increase paracellular transport of small molecules, macromolecules, or cells, (2) processes that result in dysfunction of transcellular transport, and (3) perturbations that result in disruption of normal signaling between BMECs and supporting cells, or in the secretion of soluble factors that can modulate the brain microenvironment (Table 1). Increased paracellular transport can result in entry of blood components, including hormones, proteins, and immune cells, into the brain and is sometimes referred to as “leaky brain” by analogy to leaky gut syndrome (Martinez-Ramirez et al., 2014; Haller et al., 2018). While increased paracellular transport is associated with loss of tight junctions, the rate of transport into the brain is also dependent on the glycocalyx and the basement membranes (Kutuzov et al., 2018). Overall, dysfunction can lead to changes in paracellular permeability, transport of nutrients and other essential molecules, immune cell transport, trafficking of pathogens into the brain, and loss of BMECs and/or supporting cells, which ultimately result in neuroinflammation, oxidative stress, and neurotoxicity (Hawkins and Davis, 2005; Engelhardt, 2008a,b; Abbott et al., 2010; Neuwelt et al., 2011). In general perturbations may be initiated from systemic circulation (e.g., endogenous or exogenous blood components), the brain parenchyma (e.g., neuroinflammation, toxic plaques, trauma, etc.), or intrinsically (e.g., mutations acquired by BMECs or supporting cells).

## 2. Mechanical forces

BMECs are constantly exposed to mechanical stimuli under physiological conditions. The hydrostatic pressure generated by the heart in pumping blood throughout the circulatory system exerts circumferential (hoop) and axial stresses on the endothelium, and blood flow results in a wall shear stress (Campinho et al., 2020). Furthermore, BMECs in arterioles and, to a lesser extent in capillaries, are exposed to cyclic strain due to pulsatile blood flow. BMECs can sense and transduce these mechanical cues resulting in a range of cellular responses.

### 2.1. Hypo−/hypertension

**Figure 3:**
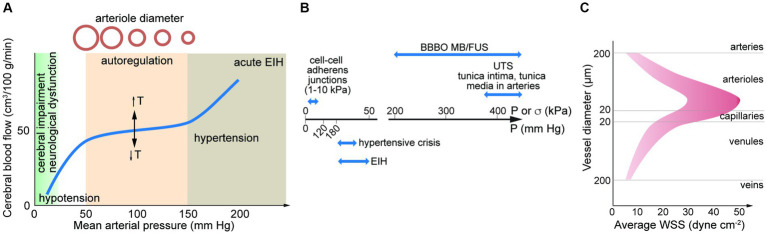
**(A)** Autoregulation results in a relatively constant cerebral blood flow over a range of mean arterial pressures (MAPs). The set-point is dependent on temperature (T). MAP can be estimated from MAP = DP + 1/3(SP – DP). SP, systolic pressure; DP, diastolic pressure. Elevated blood pressure (BP) and hypertension is defined by a BP > 180 mm Hg systolic and/or 120 mm Hg diastolic. Exercise induced hypertension (EIH) can lead to transient increases in systolic BP higher than 300 mm Hg. **(B)** Pressure associated with hypertensive crisis and EIH, and ultimate tensile stress for adherens junctions and arteries. Also shown is the force generated in MB/FUS. **(C)** Average wall shear stress along the arterio-venous axis. Data from direct measurements of flow rate and vessel diameter in the retina. UTS, ultimate tensile strength; BBBO, blood-brain barrier opening; EIH, exercise induced hypertension; MB/FUS, focused ultrasound with microbubbles.

The brain is one of the most energy intensive organs in the human body; consuming 15–20% of oxygen in circulation (Bogorad et al., 2019). Blood supply to the brain is driven by the cerebral perfusion pressure (CPP), the mean arterial pressure (MAP), and the intercranial pressure (ICP, typically ~10 mm Hg). Due to the rigidity of the skull, there is a relatively low tolerance for changes in volume induced by changes in the volume of blood or cerebrospinal fluid (Monro-Kellie hypothesis). Cerebral blood flow (CBF) autoregulation is the process by which the brain regulates changes in cerebral blood flow and hence changes in blood volume that could increase ICP. Autoregulation maintains a relatively constant blood flow (~50 mL/min/100 g of brain tissue) over a range of CPPs from about 50–150 mm Hg (Figure 3A; Lassen, 1959; Claassen et al., 2021). However, transient changes in blood pressure (milliseconds to seconds) over this range can lead to fluctuations in CBF, where reductions in CBF of more than 30–60% (the reserve capacity) result in ischemic conditions (Baron, 2001; Lidington et al., 2021).

Normal blood pressure (BP) is defined as 120/80 mm Hg (systolic/diastolic) (Flack and Adekola, 2020), although measured values vary with many factors including circadian clock, season, age, etc. (Schutte et al., 2022). Hypotension is usually defined as a BP < 90/60 mm Hg and can be caused by various factors including blood loss, dehydration, coronary artery disease, and medication. In the brain, hypotension can become dangerous if the CPP is too low to provide sufficient oxygen and glucose. Orthostatic hypotension is a reduction in BP on rising from a supine position, and is normally compensated by the autonomic nervous system. Orthostatic hypotension is characterized by a drop in BP of ≥20 mm Hg systolic and ≥ 10 mm Hg diastolic within 3 min of standing up (Joseph et al., 2017).

Increases in CPP above the range for autoregulation can result in increased ICP and increased mechanical forces on the cells of the NVU. According to current American Heart Association guidelines, elevated blood pressure is defined as 120–129 mm Hg systolic and < 80 mm Hg diastolic, stage 1 hypertension as 130–139 mm Hg systolic and/or 80–89 mm Hg diastolic, and stage 2 hypertension as >140 mm Hg systolic and/or > 90 mm Hg diastolic (Flack and Adekola, 2020). Hypertensive crisis is defined by a BP > 180 mm Hg systolic and/or 120 mm Hg diastolic (Whelton et al., 2018). Systolic BP normally increases monotonically with exercise intensity due to increased cardiac output, however, in some individuals may exhibit a hypertensive response to exercise (HRE) (Shilton et al., 2017; Moore et al., 2021). Exercise-induced hypertension (EIH) is defined as a resting BP of <140/90 mm Hg and a maximal exercise systolic BP of >210 mm Hg for males, or > 190 mm Hg for females (Schultz et al., 2013). A study of 5 power lifters reported an average peak BP of 320/250 mm Hg (MacDougall et al., 1985). However, there is no evidence for BBB dysfunction in EIH (De Silva et al., 2016).

Acute hypertensive crisis can occur under physiological (physical and psychological stress) (Kulkarni et al., 1998) and pathological conditions (e.g., TBI, seizures, and eclampsia) where autoregulation is disrupted (Schultz et al., 2013; Kim et al., 2020; Labrecque et al., 2021; Muacevic et al., 2021). Acute hypertension in healthy rats resulted in permeability to Evans blue/albumin and IgG within seconds upon increasing the MAP by ~60 mm Hg above baseline, and was reversible when the pressure returned to normal (Johansson and Martinsson, 1980; Kuang et al., 2004). Studies in animal models of acute hypertension have found BBB dysfunction occurs preferentially in post-capillary venules (De Silva et al., 2016). Studies in various animal models of chronic hypertension have found increased BBB permeability, oxidative stress, inflammation, cerebrovascular remodeling, and impairment of neurovascular coupling (Fredriksson et al., 1987; Ueno et al., 2004; De Silva et al., 2016; Setiadi et al., 2018). Increased BBB permeability has been reported to be zonation specific. For example, in a mouse model of angiotensin II hypertension, increased permeability of 3 kDa dextran was found in arterioles and venules >10 μm in diameter but not in capillaries (Santisteban et al., 2020). Some studies have suggested that the increased permeability is due to both TJ remodeling and transcytosis (Santisteban et al., 2020).

### 2.2. Focused ultrasound

Focused ultrasound has been explored for a wide range of purposes. High intensity focused ultrasound (HIFUS), with relatively long pulses (~ 1 s) at high acoustic pressures (typically 1 to 10 MPa) have been used for soft tissue ablation. HIFUS is used to ablate a wide range of solid tumors, including glioblastomas (Izadifar et al., 2020). In addition to destroying brain tissue, HIFUS can reversibly or irreversibly disrupt the BBB through disruption of cell–cell junctions (Mesiwala et al., 2002; McMahon et al., 2018). Very short pulses (~ 1 ms) at ≥20 MPa can induce cavitation, resulting in mechanical ablation of tissue.

Low energy pulsed focused ultrasound (pFUS) in conjunction with microbubbles (MBs) is used for reversible opening of the BBB. Energy from the acoustic waves induces stable (or non-inertial) cavitation which results in expansion and contraction of the MBs. These oscillations create local forces which can disrupt the endothelium, either forming pores in cell membranes or disrupting cell–cell junctions. The increase in permeability has been used in clinical trials to deliver therapeutics to treat diseases of the brain (Lipsman et al., 2018; Idbaih et al., 2019; Mainprize et al., 2019). The magnitude of the effect is dependent on ultrasound frequency, excitation pressure, and pulse duration (typically <1 ms). The acoustic pressure needed for BBB opening by pFUS/MB is typically in the range 100–500 kPa. This is approximately 10-fold higher than systolic blood pressures (120 mm Hg = 16 kPa) and the threshold for hypertensive crisis (180 mm Hg = 24 kPa) (Figure 3B).

The MBs are gas-filled, lipid coated microbubbles, similar to liposomes, and are typically 2–5 μm in diameter, slightly smaller than the diameter of capillaries in the brain (about 7–8 μm in humans) (Song et al., 2018). To enhance stability, MBs are filled with a high molecular weight gas with low aqueous solubility, such as perfluorocarbons. Under FUS excitation, the increase in MB diameter can be up to about 10-fold, depending on the diameter and the stiffness of the lipid shell and the viscosity of the fluid (Roovers et al., 2019). pFUS/MB results in transient pore formation in cell membranes and disruption of cell–cell junctions, both of which can result in changes in normal cell function, although these effects have not been widely studied.

In capillaries, BMECs exhibit a bamboo-like structure, and hence disruption of cell–cell junctions is more likely to occur when a bubble is close to one of the junctions. Consequently, the increase in permeability is thought to be associated with focal leaks that are randomly located in the region where the ultrasound is focused (Roovers et al., 2019). Dependent on the conditions, the defects at cell–cell junctions may allow entry of entities from small molecules, such as trypan blue, to large molecules, such as antibodies and plasmid vectors (Kinoshita et al., 2006; Kovacs et al., 2017; McMahon and Hynynen, 2017). Higher doses can result in entry of cells from circulation, as evidenced by local hemorrhage (McDannold et al., 2008; McMahon and Hynynen, 2017). Imaging of gadolinium leakage into the brain has shown that BBB opening occurs immediately after pFUS treatment (Hynynen et al., 2005) and persists for a few hours to a few days (Kovacs et al., 2017; Song et al., 2018) depending on the size of microbubbles. Studies have also shown that pFUS treatment can induce inflammation and immune responses that persist for at least 6 days post-sonication (Kovacs et al., 2017; McMahon and Hynynen, 2017). Whether pFUS-induced barrier changes and inflammation/immune responses act synergistically or antagonistically to treat brain diseases is not yet fully understood.

### 2.3. Traumatic brain injury and concussion

Traumatic Brain Injury (TBI) can be caused by direct impact, sudden or rapid acceleration and deceleration, penetrating injury, or blast injury. In animal TBI/concussion models, brain damage occurs with mechanical stresses from 100–500 kPa (Courtney and Courtney, 2007; Courtney and Courtney, 2015; Guley et al., 2016). In TBI, the impact of brain tissue on the inside of the skull can cause swelling, contusions, diffuse axonal injury, and disruption of blood vessels. The primary physical impact can cause significant displacement between different structures in the brain leading to shear, tensile, and compressive forces (Sahyouni et al., 2017), and resulting in structural damage to blood vessels and microhemorrhage. TBI and concussion can lead to disruption of the BBB via the primary physical injury as well as secondary responses such as inflammation and oxidative stress (Shlosberg et al., 2010; Sahyouni et al., 2017). In animal models of concussion/TBI, BBB disruption is typically biphasic with an early acute phase at 4–6 h and a delayed chronic phase starting 3–7 days after injury (Baskaya et al., 1997; Shlosberg et al., 2010; Xiong et al., 2013). Neovascularization is typically observed within 48 h after TBI and vascular remodeling is seen after 4 days (Prakash and Carmichael, 2015). Functional changes in transport have also been observed: in a rat TBI model, increased endothelial caveolae-mediated transcytosis was observed during the first 4 days following TBI with a subsequent decrease in expression of claudin-5 (Nag et al., 2009).

In humans, BBB opening associated with concussion can result in leakage of plasma proteins, such as fibrinogen and IgG (Johnson et al., 2018). MRI studies have found correlations between the amount of BBB leakage and serum levels of matrix metalloproteinase-7 (MMP-7) and S100B (O’Keeffe et al., 2020; Nichols et al., 2021). BBB leakage may persist for days to weeks, and long lasting effects of TBI include increased risk of neurodegenerative disease (Shlosberg et al., 2010; Prakash and Carmichael, 2015). BBB disruption is also a hallmark of Chronic Traumatic Encephalopathy (CTE), the consequence of repeated TBI, a brain trauma common in contact sports such as boxing and American football. Patients with CTE exhibit cerebral edema and enlarged perivascular spaces (Sweeney et al., 2018).

### 2.4. Wall shear stress

Blood flow results in a wall shear stress (WSS) on endothelial cells. WSS is dependent on the flow rate, blood viscosity, and vessel diameter. From Poiseuille’s law, the WSS (τ) in straight sections of cylindrical vessels under laminar flow is given by:


$$\tau =\frac{32\eta Q}{\pi d^{3}}$$

where *Q* is the flow rate, η is the blood viscosity (dependent on shear rate but typically 2–4 mPa·s), and *d* is the diameter of the vessel lumen.

Accurate data on flow rate in the human cerebrovasculature is limited. From measurements of vessel diameter and blood flow in microvessels in the human brain using MRI or in the human retina or conjunctiva using optical techniques, the WSS can be estimated from Poiseuille’s Law. While viscosity increases with increasing shear rate, we assume η=2.2 mPa·s (cP). From these studies, the mean WSS is determined to be ~10–50 dyne cm^−2^ in arterioles (20–180 μm diameter), 10–30 dyne cm^−2^ in capillaries (9–19 μm diameter), and 5–20 dyne cm^−2^ in venules (30–300 μm diameter) (Figure 3C; Riva et al., 1985; Koutsiaris et al., 2007; Wang et al., 2007, 2009; Shahidi et al., 2010). A meta study of hemodynamics in the human common carotid artery reported a mean WSS of 11–13 dyne cm^−2^ (Reneman et al., 2006). These values should be considered approximate based on the limited applicability of Poiseuille’s Law. Average values of WSS in this range are important for maintenance of EC quiescence and NVU homeostasis. In arteries, values below this range along with disrupted flow patterns can lead to a range of pathological responses. High mean WSS (> 25 dyne cm^−2^) is less well studied, but is also considered to be pathological. From *in vitro* studies of monolayers in 2D, ECs undergo a morphological transformation from cobblestone to spindle-like under WSS of 5–20 dyne cm^−2^ (DeStefano et al., 2017).

### 2.5. Cellular level responses to mechanical stress

At the cellular level, the circumferential and axial stresses exert a tensile force on the cell–cell junctions. The ultimate tensile strength for cell–cell junctions between two isolated cells is in the range 1–10 kPa (Charras and Yap, 2018). However, the supporting cells, extracellular matrix (ECM), and basement membrane in tissues significantly increase the strength of the vessel wall. The ultimate tensile strength of the tunica intima and tunica media of large arteries is around 400 kPa, and for the tunica externa is >1,000 kPa (Camasao and Mantovani, 2021; Mishani et al., 2021). Changes in circumferential stress can arise from physical perturbations (e.g., acute/chronic high blood pressure, microbubble distortion in FUS/MB, TBI), or from exposure to biochemical perturbations that result in increased tensile stress on cell–cell junctions (e.g., osmosis) or cell loss from the lumen.

**Figure 4:**
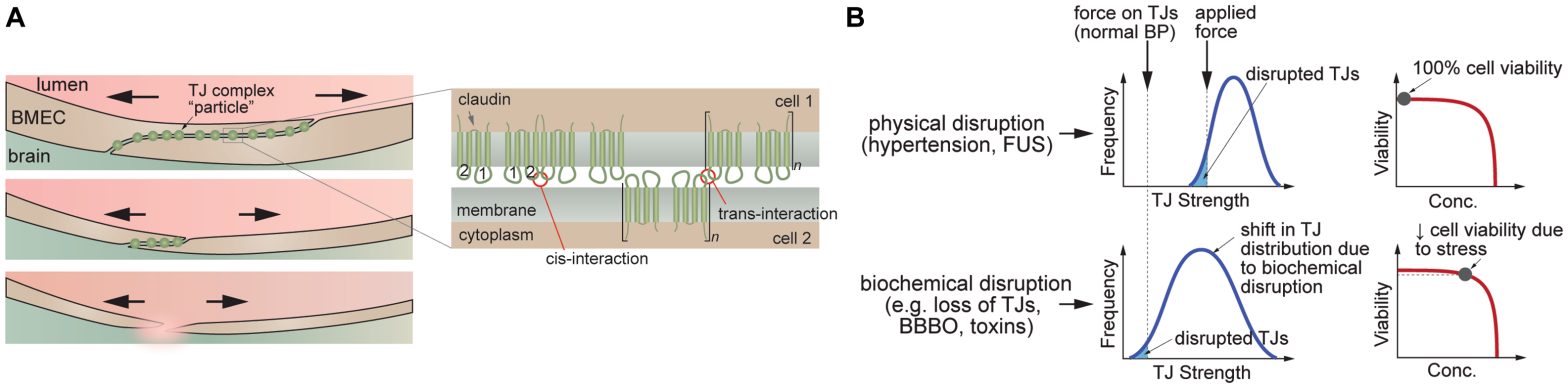
Disruption of tight junctions (TJs). **(A)** Schematic illustration of TJ disruption. Tensile forces result in disruption of the cell–cell junction and reduction in the number of TJs. **(B)** TJ disruption can be induced by tensile forces at cell–cell junctions (e.g., hypertension, FUS) or downregulation of TJ proteins leading to a decrease in the number of pinning points at the cell–cell junction (e.g., decrease in heterotypic interactions between claudin-5 extracellular domains). **(C)** Schematic illustration of trans-interactions between of claudin-5 extracellular domains that contribute to TJs.

If the circumferential stress on the endothelium is sufficiently large, then junction disruption can lead to an increase in paracellular transport (Figure 4A). Since there is expected to be a range of TJ tensile strengths in a monolayer (Figure 4B), the weakest cell–cell junctions are more likely to be disrupted, leading to spatially localized focal leaks. In contrast, biochemical stress can result in a shift in the distribution of TJ strength that results in junction disruption at physiological pressures (Figure 4B). For example, a biochemical stress can cause a decrease in cell viability and hence loss of BMECs that is not immediately compensated by the remaining cells in the endothelium, resulting in local increases in paracellular permeability. If the decrease in viability is small, the endothelium may be repaired. In another example, biochemical stress (e.g., osmotic stress) may cause cells to shrink, resulting in both a decrease in cell viability and increased tensile stress on the cell–cell junctions. Indeed, chemical methods for reversible blood-brain barrier opening rely on the delicate balance between biological stress and recovery.

## 3. Temperature

The average core body temperature (*T*_c_) of healthy individuals is around 36°C (Mackowiak et al., 1992; Mackowiak and Worden, 1994; Sund-Levander and Grodzinsky, 2009; Protsiv et al., 2020). The average temperature of the brain is typically 1–2°C higher than *T*_c_ due to its high metabolic rate (Bain et al., 2015). Recent studies suggest that brain temperature varies with brain region and age, with temperatures as high as 40°C measured in the thalamus of healthy adults (Rzechorzek et al., 2022). The average brain temperature shows diurnal cycles, with the lowest temperature at night when CBF is highest, although these cycles are compromised with aging (Rzechorzek et al., 2022).

**Figure 5:**
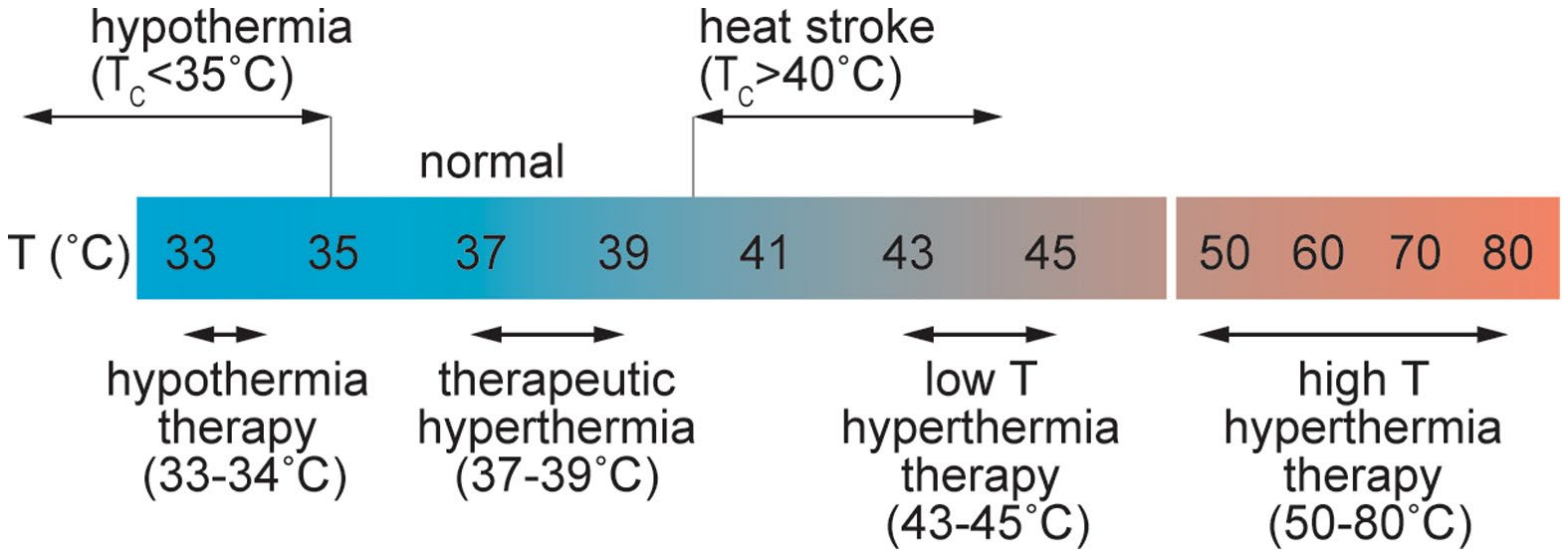
Schematic illustration of the range of perturbations in temperature associated with pathological and therapeutic conditions.

Common causes of elevated temperature include fever, passive heating, and exercise. Heat stress is usually defined by an increase in *T*_c_ up to 40°C (Figure 5; Bain et al., 2015), and heat stroke is associated with core temperatures above 40.5°C (Bouchama and Knochel, 2002). Heat stroke can cause symptoms including delirium, convulsions, and coma and has been defined as hyperthermia with a systemic inflammatory response leading to multi-organ failure and encephalopathy (Bouchama and Knochel, 2002). Heat stroke results in elevated systemic levels of the inflammatory cytokines TNF-α, and IL-1β along with IL-6 and IL-10 (Bouchama and Knochel, 2002).

Core temperatures of 46–60°C are associated with irreversible cellular damage where the level of damage is often proportional to the exposure time. At the cellular level, *in vitro* studies have shown that cell death can occur at temperatures above 42°C. In many cases cell viability decreases approximately exponentially with time at a given temperature but is dependent on the cell type (Harmon et al., 1990; Thomsen, 1991; Dewhirst et al., 2003). Protein unfolding, which occurs at 50–90°C, also results in irreversible cell damage. Temperatures in excess of 100°C are associated with water vaporization, and temperatures greater than around 300°C result in tissue ablation and vaporization (Thomsen, 1991).

Therapeutic hyperthermia and hyperthermia therapy exploit systemic or local increases in temperature for treatment of various diseases. Therapeutic hyperthermia is usually associated with increases in *T*_c_ of 1–1.5°C. Small increases in temperature result in increased blood flow and is a potential tool for management and prevention of cardiovascular disease (CVD) (Tinken et al., 2009). However, higher temperatures can induce increased BBB permeability. In a rat model of whole body hyperthermia, young animals exposed to 38°C for 4 h showed BBB disruption and microhemorrhage (Sharma and Dey, 1986). Brain penetration of Evans blue/albumin was observed in regions of the cortex, cerebellum, hypothalamus, and thalamus, providing evidence for increased paracellular transport (Sharma and Dey, 1986). No disruption was observed following shorter exposure, and only mild disruption was found in adult mice demonstrating that both age and dose are important in the response to hyperthermia. BBB dysfunction was associated with increases in *T*_c_ of 3.51 ± 0.41°C. Exposure of rats to temperatures ≥39°C, or *T*_c_ values in excess of 42°C, frequently resulted in animal death (Sharma and Hoopes, 2003).

Various methods of local heating (laser, ultrasound, radio frequency, microwave) are used in clinical practice, usually for treatment of solid tumors (Thomsen, 1991; Dewhirst et al., 2003; Rieke and Butts Pauly, 2008). Hyperthermia therapy is usually associated with local induction of cell death and hence a local increase in BBB permeability is a consequence of endothelial cell loss. In the low temperature regime of hyperthermia therapy, local exposure to temperatures of 43–48°C for 10–60 min can induce cell death or sensitize cells to chemotherapy or radiation therapy. In the high temperature regime, short exposures to temperatures from 50–80°C induces tissue necrosis. Laser interstitial thermal therapy (LITT) is used for treatment of various types of brain tumors and epilepsy (Patel and Kim, 2020). Local heating in the brain can also result in increased permeability of the BBB, which has been exploited in enhanced drug delivery for treatment of brain tumors. In a monkey model, extravasation of Evans blue/albumin was observed in a region of the brain heated to 43°C after 60 min. Minimal leakage was observed 3 days after treatment (Nakagawa et al., 1994).

Hypothermia is associated with core body temperatures lower than 35°C. Nontherapeutic or accidental hypothermia is most commonly associated with trauma patients and is potentially fatal (Rosli et al., 2020). Therapeutic hypothermia therapy (THT) has been studied for treatment of various brain injuries including ischemic encephalopathy and stroke (Yenari and Hemmen, 2010; Kuczynski et al., 2020). THT for brain injury can be achieved through systemic cooling or selective cooling of the head by surface cooling or intra-arterial infusion of cold saline, and has been extensively studied for protection against acute ischemic stroke. Animal studies suggest that inducing temperatures of 32–34°C for 12–24 h can provide neuroprotection through increased BBB function, decreased inflammatory responses, reduced apoptosis, and decreased cerebral metabolism (Busto et al., 1987; Dumitrascu et al., 2016; Caroff et al., 2020; Kuczynski et al., 2020; Wu L. F. et al., 2020). Trials in humans have demonstrated selective cooling in the brain by inter-arterial perfusion of hypothermic saline at 4–17°C (Choi et al., 2020; Wu D. et al., 2020).

## 4. Electromagnetic radiation

**Figure 6:**
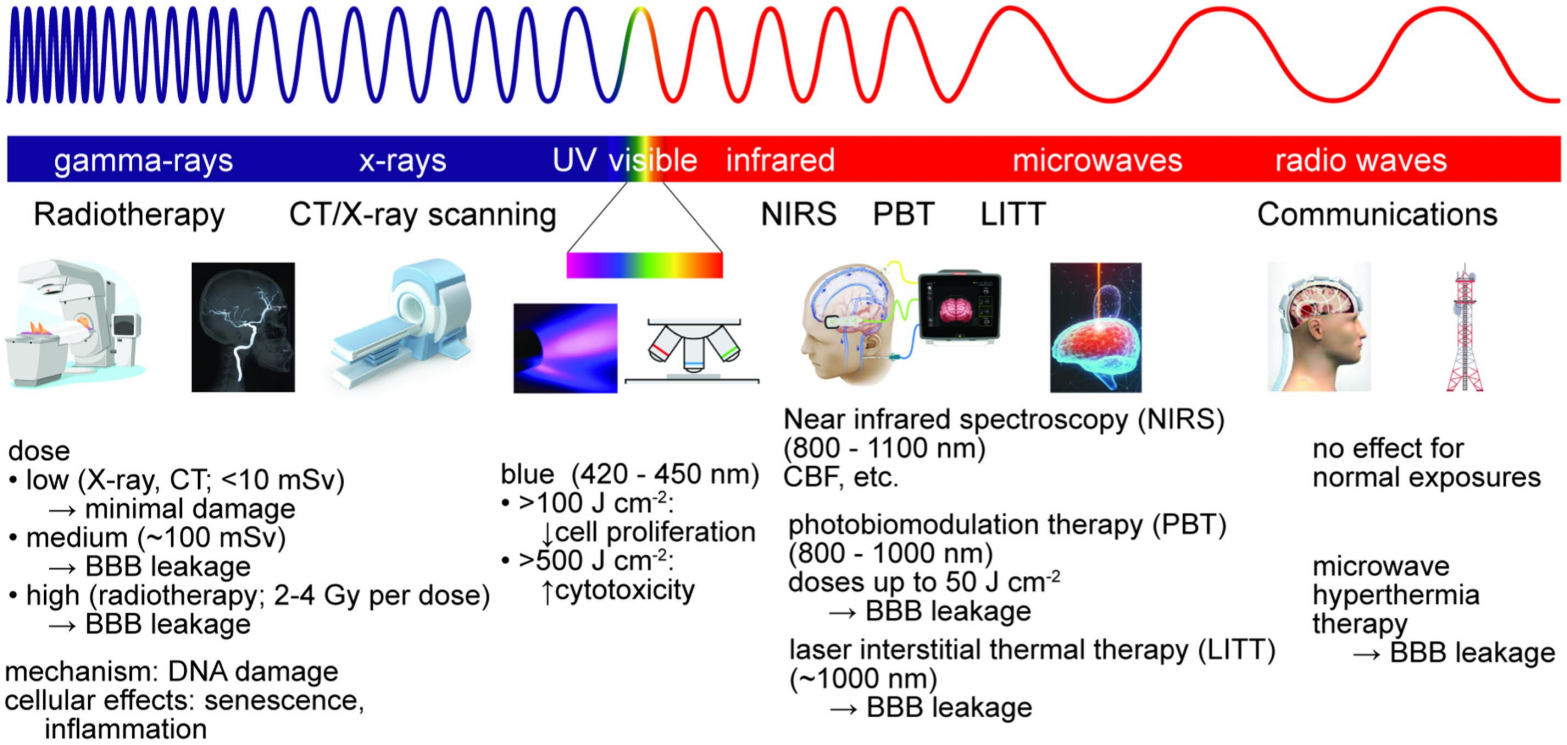
Electromagnetic radiation is used for brain imaging and therapy. Non-ionizing radiation is used for imaging, hyperthermia therapy, and photobiomodulation therapy (PBT). Ionizing radiation is used for imaging and radiation therapy, typically via DNA damage. At sufficiently high doses, exposure to ionizing or non-ionizing radiation can lead to reversible or irreversible BBB damage.

To interact with brain tissue and impact BBB function, EM radiation must penetrate the skin, skull, and meninges. Damage to brain tissue is dependent on the wavelength and amount of energy absorbed, as well as the consequences of absorption in cells or other tissue components (e.g., heating, induced electronic transitions, generation of toxic species) (Figure 6). Damage to the BBB may be due to energy absorption in the cells of the BBB (heating) or a bystander effect from dysfunction induced in the surrounding brain parenchyma. Electromagnetic radiation includes ionizing (gamma ray and X-ray), ultraviolet, visible, infrared, microwave, and radio waves, spanning wavelengths from 1 pm (gamma rays) to 1 m (radio waves). These types of radiation are ubiquitous in daily life, for example solar irradiation (e.g., ultraviolet, visible and infrared) and communications (e.g., microwave or radio waves), and/or are used in specific applications, such as clinical diagnosis or therapy (e.g., infrared, gamma, or X-ray).

### 4.1. Ionizing radiation

The most likely sources of brain exposure to high levels of ionizing radiation are associated with clinical diagnosis or treatment (e.g., CT or X-ray scans, radiotherapy) (Lin, 2010). The SI unit for absorbed dose is the Gray (Gy), defined as energy (joules) per kilogram of tissue. The health effects of ionizing radiation are defined by the equivalent dose in sieverts (Sv), which also has units of J kg^−1^. A dose of 1 Sv represents a 5.5% risk of developing cancer, and an effective dose of 4–5 Sv in a short duration is considered to have a 50% risk of death within 30 days (LD50/30) (Huang et al., 2009). Doses for conventional X-ray scans vary from 0.01–1 millisieverts (mSv), whereas doses for CT scans vary from 2–10 mSv (Mettler et al., 2000; Wiest et al., 2002; Huang et al., 2009).

Whole brain radiation therapy (WBRT) is widely used to treat brain metastases, and primary brain tumors after resection (Borras et al., 2015). To minimize side effects in surrounding tissue, radiation therapy is usually administered in a series of small doses, or fractions, to improve outcomes. For example, the standard radiation therapy dose regimen for high grade gliomas is 60–65 Gy delivered in 1.8–2.0 Gy fractions over 6 weeks, and for cerebral metastases is 20–30 Gy in 3–4 Gy fractions over 2 weeks (RCR, 2019). Radiation therapy of a single metastasis may involve a single dose of 15–24 Gy.

Radiation-induced decline in cognitive function has been reported in patients who have received WBRT (Li et al., 2008; Welzel et al., 2008), as well as in animal models (Pena et al., 2000; Greene-Schloesser et al., 2012; Warrington et al., 2012, 2013; Sándor et al., 2014; Cekanaviciute et al., 2018). MRI studies of patients with either primary or metastatic brain tumors have found dose-dependent leakage of contrast agent into the brain immediately following radiation therapy (acute injury phase), indicating an increase in BBB permeability (Curnes et al., 1986; Cao et al., 2009). Although barrier function is usually restored post treatment, correlations have been found between BBB permeability and loss of cognitive function, indicating that BBB permeability may be a predictor of radiation-induced neurocognitive dysfunction (Cao et al., 2009). While transient BBB permeability suggests a route for delivery of drugs (e.g., chemotherapeutics), BBB leakage is inconsistent. For example, individuals with metastatic brain cancer, glioblastoma, and medulloblastoma display heterogeneous BBB permeability (Arvanitis et al., 2020). Triple negative and basal-type metastatic brain cancer often results in increased BBB permeability, whereas HER2-positive metastatic brain cancer is less likely to show increased BBB permeability (Yonemori et al., 2010). These differences are likely related to differences in tumor/vessel interactions in the local microenvironment (Silvestri et al., 2020).

The primary mechanism of tumor cell death in radiation therapy is via DNA damage. However, the effect on normal tissue is highly variable among patients. Several studies in animal models have shown a significant loss of spinal cord ECs and BMECs following irradiation (Ljubimova et al., 1991; Pena et al., 2000; Sándor et al., 2014). In addition, radiation doses as low as 100 mGy resulted in leakage of Evans blue/albumin into the brain in adult mice 1 week post-irradiation (Sándor et al., 2014). *In vitro* studies have found that irradiation of BMECs (2–8 Gy) induces cellular senescence which impairs the angiogenic response to hypoxic conditions (Ungvari et al., 2013). Based on these results, it has been proposed that ionizing radiation results in loss of BMECs leading to local tissue hypoxia and a decrease in vascular density, and that repair is inhibited by cellular senescence which impairs the angiogenic response to hypoxic conditions (Ungvari et al., 2013). This is similar to the vascular hypothesis for radiation damage in the brain, which posits that damage and loss of BMECs along with local release of inflammatory cytokines leads to necrosis and increased edema and microhemorrhage (Walker et al., 2014). Furthermore, ionizing radiation can induce an inflammatory response in BMECs, resulting in adhesion and transmigration of circulating immune cells, leading to loss of barrier function, as well as generation of reactive oxygen species (Olschowka et al., 1997; Sharp et al., 2003; Yuan et al., 2003; Lumniczky et al., 2017).

### 4.2. Space radiation

On Earth, humans are exposed to about 3 mSv of radiation a year from natural sources and the low level of cosmic rays that penetrate the atmosphere (Shea and Smart, 2000). In space, humans are exposed to cosmic ionizing radiation at levels substantially higher than on Earth (Nelson, 2016). For astronauts on the International Space Station, exposure is around 300 mSv per year, and exposure in interplanetary space can be more than 700 mSv per year (Cucinotta and Durante, 2006). In spaceflight, radiation exposure is coupled with microgravity, which is known to influence brain structure and function, resulting in increased intercranial pressure and altered cerebral blood flow. Therefore, the combined effects of radiation exposure and microgravity may be compounded. Despite the importance for human health in space, very little is known about the combined influence of microgravity and cosmic radiation on BBB function (Cekanaviciute et al., 2018).

### 4.3. Ultraviolet, visible, and infrared radiation

Humans are exposed to UV, visible, and infrared radiation during normal daily life. Levels of exposure to solar irradiation are not sufficient to penetrate the skull, and hence exposure is usually not an issue for brain health. The average solar irradiance at sea level is about 100 mW cm^−2^, corresponding to an energy deposition of 100 mJ cm^−2^ for a 1 s exposure (Solanki et al., 2013).

Studies in cadaver skulls and *in vitro* models of brain tissue have shown significant transcranial deposition of infrared energy at certain wavelengths in the range from 810–1,100 nm (Jagdeo et al., 2012; Cassano et al., 2019). Transcranial near infrared spectroscopy (NIRS) relies on the penetration of near IR radiation from an array of LEDs to detect changes in blood volume, blood oxygenation, and cerebral blood flow in cortical tissue (Chen W. L. et al., 2020). Due to the strong absorption in water and hemoglobin, the penetration depth is typically limited to about 2 cm. The LEDs commonly used for in NIRS typically have incident power densities of 10–20 mW cm^−2^ and use relatively short pulses (< 1 s).

Although the brain is not usually exposed to visible and ultraviolet radiation, absorption of visible light in tissues can generate heat or toxic molecules. Therefore, visible and UV radiation is a potential source of cell damage when imaging *in vitro* BBB models. Not surprisingly, most of our knowledge is based on exposure of skin cells to visible and ultraviolet irradiation. Exposure of human keratinocytes or dermal microvascular endothelial cells to red and infrared light (632–940 nm) had no effect on cell viability, whereas exposure to blue light (412–426 nm) at high fluences (66–100 J cm^−2^) or at 453 nm with very high fluences (>500 J cm^−2^) resulted in cytotoxicity (Liebmann et al., 2010). Exposure of endothelial cells or keratinocytes to blue light at nontoxic fluences resulted in a reduction of proliferation of up to 50% in a dose dependent manner (Liebmann et al., 2010).

### 4.4. Laser therapy

Brain photobiomodulation (PBM) therapy involves exposure to near-infrared (NIR) light (800–1,000 nm) (Salehpour et al., 2018). LEDs with power densities of 10–20 mW cm^−2^ are used to irradiate the brain for durations of minutes to hours, corresponding to doses of up to 50 J cm^−2^ (Hamblin, 2016). Near-IR radiation absorbed by the cytochrome c oxidase (COX) complex within mitochondria stimulates the electron transport chain which produces ATP. PBM can also produce NO and modulate ROS production. Studies have shown that PBM can increase gamma brain oscillations (Gonzalez-Lima and Barrett, 2014). Other regions of the brain, such as the prefrontal cortex, can absorb IR radiation via intranasal illumination (Pitzschke et al., 2015).

High energy laser irradiation can produce high temperatures in tissues, resulting in cell apoptosis and tissue necrosis. MRI-guided laser interstitial thermal therapy (LITT) is an emerging minimally invasive technique to treat primary and metastatic brain tumors and focal epilepsy (Holste and Orringer, 2020; Patel and Kim, 2020). A laser catheter is surgically guided into the tumor and wavelengths around 1,000 nm are used to generate local temperatures of 43–45°C. Guided by MRI or other imaging techniques, laser thermal therapy it can be used to kill cancer cells, but since the resolution is limited, can also induce disruption of the BBB resulting in cerebral edema and intracranial hemorrhage (Hawasli et al., 2014; Holste and Orringer, 2020). The main limitation of this method is the difficulty in controlling the temperature and avoiding adverse effects beyond the target zone. Studies in mouse models have shown that laser thermal therapy can induce solute extravasation or vessel occlusion dependent on the energy and doses (Nishimura et al., 2006). A clinical study of individuals with recurrent glioblastomas treated with LITT found that BBB permeability of MRI contrast agent peaked within 1–2 weeks after therapy and resolved within 4–6 weeks (Leuthardt et al., 2016). This increase in BBB permeability can be used to deliver drugs and other therapeutics alongside laser ablation of cancer cells, enabling combination therapy that may increase treatment efficacy (Patel et al., 2020; Patel and Kim, 2020; Lerner et al., 2022).

### 4.5. Microwaves and radio waves

Microwaves (300 MHz – 300 GHz) and radio waves (3 kHz – 300 MHz) are ubiquitous in communications and appliances, however, the energy of electromagnetic radiation in this frequency range is relatively low and cannot directly cause DNA damage. There is no conclusive evidence that normal daily exposures cause cancer or other pathologies, however, the effect of exposure in tissue is dependent on wavelength, power, and duration. The primary effect of tissue absorption of electromagnetic radiation in this frequency range is heating. At sufficiently high doses, microwaves and radio waves can induce tissue damage. For example, microwave hyperthermia therapy is used in the treatment of several cancers including brain cancer.

## 5. Hypoxia

**Figure 7:**
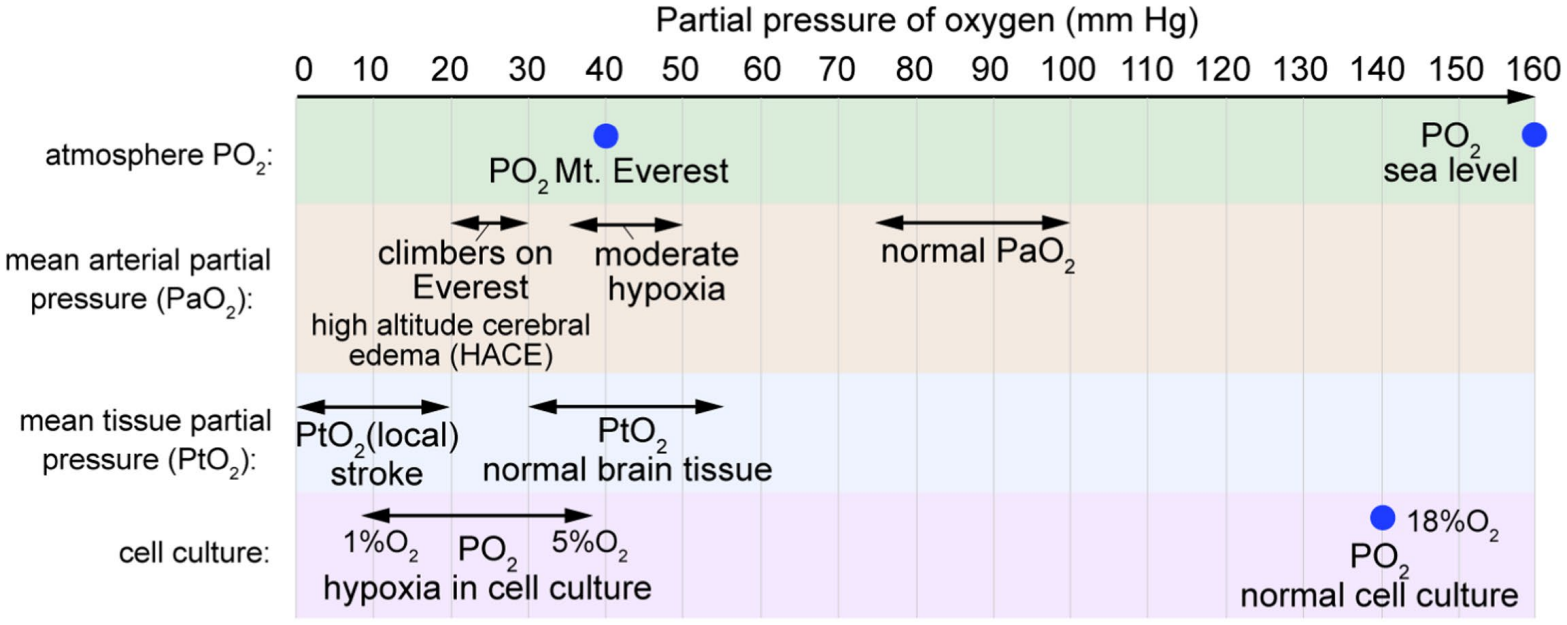
Partial pressure of oxygen. The mean arterial partial pressure of oxygen (PaO_2_) is typically 75–100 mm Hg. The mean arterial partial pressure of oxygen in brain tissue (PtO_2_) is typically 30–55 mm Hg. In normal cell culture, cells are maintained in humidified atmospheric air at 37°C with 5% CO_2_, resulting in PO_2_ values of 18–19% O_2_ (~140 mm Hg).

Hypoxia is a condition in which oxygen demand in tissues or organs exceeds supply. At sea level, the partial pressure of oxygen (PO_2_) is about 160 mm Hg (Figure 7). Oxygen is supplied to organs via circulation, where the normal arterial oxygen partial pressure is in the range 75–100 mm Hg, decreasing to 30–40 mm Hg in veins (Ortiz-Prado et al., 2019). The average oxygen partial pressure in healthy human brain tissue is reported to be in the range 30–50 mm Hg (Carreau et al., 2011; Ortiz-Prado et al., 2019). However, tissue oxygen partial pressure in the cortex can increase to 140–350 mm Hg for subjects breathing 100% O_2_ (Meixensberger et al., 1993). Reduced O_2_ levels in the brain can occur due to decreased CBF or CPP, decreased partial pressure of oxygen in circulation (hypoxemia), decreased hemoglobin, increased metabolism, or local occlusion of vessels (e.g., ischemia).

Following ischemic stroke, blood flow is reduced to a local region of the brain, usually due to an occlusion. Ischemia initiates a cascade of pathological events, including neuronal cell death, production of reactive oxygen species (ROS), metabolic changes, inflammation, BBB dysfunction, and neuronal cell death (Durukan and Tatlisumak, 2007; Krueger et al., 2015; Prakash and Carmichael, 2015). While the timing remains somewhat controversial, BBB dysfunction includes an increase in paracellular permeability by cell loss or loss of tight junctions, and/or an increase in transcellular transport by upregulating transcytosis (Plateel et al., 1997; Fischer et al., 2000). Claudin-5 and occludin have been shown to be degraded by MMP-9, which is highly expressed during ischemia (Bauer et al., 2010). Recent studies have found an increased prevalence of endocytic vesicles at early time points after ischemia, which suggests that transcellular transport may contribute to early BBB dysfunction (Haley and Lawrence, 2017).

*In vitro* studies of BMECs exposed to oxygen/glucose deprivation (OGD) have demonstrated phosphorylation and delocalization of TJ proteins, including claudin-5 and occludin. These changes appear to be downstream of hypoxia-inducible factor-1 (HIF-1) and may be mediated in part by increased secretion of VEGF (Engelhardt et al., 2014; Kokubu et al., 2017; Page et al., 2019). While astrocytes and pericytes appear to be more resilient than BMECs to OGD-induced apoptosis, these cells have been shown to respond to OGD by secreting MMPs (Lu et al., 2009) or by modulating BMEC barrier function through the secretion of growth factors, such as VEGF (Schmid-Brunclik et al., 2008), or cytokines, including IL-1β (Argaw et al., 2006). Astrocytes and pericytes also undergo structural changes following ischemic stroke, with swelling and detachment of astrocyte end-feet, and pericytes either migrating away from the endothelium, or constricting and preventing reperfusion (Liu et al., 2012).

While hypoxia is a key BBB stressor during ischemic stroke, it is difficult to distinguish BBB dysfunction caused by hypoxia from other stressors including glucose depletion, loss of shear stress, and the accumulation of waste products, each of which can independently contribute to BBB dysfunction (Pearigen et al., 1996).

**Figure 8:**
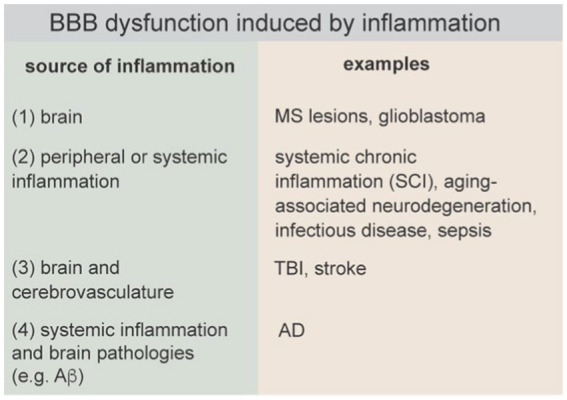
BBB dysfunction induced by inflammation can be initiated in the brain, by peripheral or systemic inflammation, simultaneously in the brain and cerebrovasculature, or systemically and amplified by brain pathologies.

At sea level, PO_2_ is approximately 160 mm Hg, whereas at the summit of Mount Everest (8,848 m), PO_2_ is 43 mm Hg (Figure 8). Arterial PO_2_ values of 20–30 mm Hg have been reported in climbers at 8,400 m on Mt. Everest (Grocott et al., 2009). Acute mountain sickness (AMS) affects up to 40 percent of hikers rapidly ascending to 3,000 m (9,800 ft) (Hackett and Roach, 2001), causing symptoms including headache, nausea, vomiting, fatigue, and sleep disruption (Baneke, 2010). After 3–5 days of acclimatization, cerebral blood flow returns to normal and AMS symptoms resolve. Severe cases can lead to high altitude cerebral edema (HACE) or high altitude pulmonary edema (HAPE), either of which can be life threatening. In severe AMS cases that progress to HACE, cytotoxic edema causes brain swelling, intracranial pressure increases further, and BBB permeability may increase (Baneke, 2010; Lawley et al., 2014; Davis and Hackett, 2017). Acute exposure to severely reduced PO_2_ results in loss of consciousness (e.g., depressurization of an aircraft), however, more gradual changes result in adaptations such as increased vascular density that can occur over several weeks (Bogorad et al., 2019).

## 6. Exogenous factors

Exogenous compounds that can induce BBB dysfunction include those administered to enhance drug delivery (e.g., hyperosmotic agents, vasoactive compounds, alkylglycerols (AKGs), sodium caprate, and membrane active peptides), those consumed recreationally (e.g., alcohol), and those associated with unintentional exposure (e.g., toxins) (Figure 1). These compounds display diverse mechanisms by which BBB dysfunction can occur following acute and/or chronic exposure.

### 6.1. Chemical blood-brain barrier opening

Hyperosmolarity causes a wide range of cellular changes including cell shrinkage, induction of apoptosis, DNA damage, and heat shock response (Alfieri and Petronini, 2007). Systemic intravenous (IV) administration of hyperosmotic agents, particularly mannitol, is widely used in the clinic to reduce cerebral edema. However, exposure to mannitol at higher concentrations can induce BBB opening (BBBO) (Rapoport et al., 1972, 1980; Rapoport, 2000; Linville et al., 2019). Since the threshold is near the solubility limit, BBBO is achieved by intra-arterial (IA) delivery (Rapoport et al., 1972, 1980; Rapoport, 2000). The mechanism of mannitol-induced BBBO is related to the increased tensile stress on cell–cell junctions induced by cell shrinkage and vasodilation (Rapoport et al., 1972, 1980; Rapoport, 2000). Recent work suggests that it is the weakest junctions that are susceptible to disruption, leading to transient focal leaks in the endothelium (Linville et al., 2019).

BBBO involves inducing sufficient cell stress to disrupt local cell–cell junctions but not the extent that the process is irreversible. Therefore, BBBO is a dynamic process with a spatio-temporal response that depends on dose and location in the brain. Increased paracellular transport is almost instantaneous after dosing (Chu et al., 2018), and hence therapeutics are commonly administered intra-arterially immediately following hyperosmotic agents as a bolus dose. Depending on administration conditions, barrier function can be restored within 5–10 min after administration (Rapoport et al., 1980; Houthoff et al., 1982). Osmotic BBBO has been used for delivery of chemotherapeutics, antibodies, nanoparticles, and gene vectors into the brain (Muldoon et al., 1995; Doolittle et al., 2000; Burkhardt et al., 2012; Lesniak et al., 2019), but has not achieved widespread clinical use in humans, at least partially due to challenges with reproducibility.

Various vasoactive compounds have been explored for transient BBBO, including bradykinin (an agonist for bradykinin receptor B2), bradykinin analogs (labradimil, retro-inverso bradykinin), adenosines (agonists for adenosine receptors) and adenosine analogs (e.g., regadenoson), endothelin-1 (an agonist for endothelin receptors A and B, ETA and ETB), C type natriuretic peptide (CNP) (an agonist for natriuretic peptide receptors B and C (NPR-B and NPR-C)), among others. Of these, bradykinin receptor is the most well-studied. Bradykinin and its synthetic analog (labradimil) are vasoactive peptides that activate β_2_ receptors constitutively expressed on BMECs (Borlongan and Emerich, 2003). Bradykinin is an endogenous peptide which at physiological concentrations does not disrupt the BBB. However, intracarotid injection of 4 μM labradimil induced leakage of fluorescein into the cortex in a cat model, but not 20 kDa dextran or albumin (Unterberg et al., 1984). Injection of the β-receptor agonist isoproterenol into the brain parenchyma reduced fluorescein leakage, confirming the role of receptor mediated uptake in inducing BBB leakage. EM analysis of vessels following intrajugular injection of labradimil in mice found significant tight junction penetration of lanthanum tracer but negligible changes in the number of vesicles or endocytic pits, supporting paracellular leakage due to disruption of tight junctions (Sanovich et al., 1995). In addition, *in vitro* studies have shown that activation of β_2_ receptors on rat BMECs causes calcium influx (Doctrow et al., 1994). IV administration of labradimil along with an analgesic in a rat model resulted in a two-fold increase in analgesic effect (Emerich et al., 1998). Subsequent studies of labradimil administration for transient BBBO and delivery of chemotherapeutics for treatment of brain cancer in animal models (Inamura and Black, 1994; Fike et al., 1998; Emerich et al., 1999, 2000, 2001) led to a clinical trial in humans (Cloughesy et al., 1999; Gregor et al., 1999; Prados et al., 2003). However, therapeutic effects have been inconsistent, resulting in less interest in vasoactive peptides as a means for BBBO.

Membrane active peptides (MAPs) are a new classes of compounds and have potential to induce BBBO (Sarkar et al., 2014; Meng et al., 2017; Aasen et al., 2019). Compared to hyperosmotic agents, which have low molecular weights, peptide-based strategies have customizable physiochemical properties (i.e., charge, hydrophobicity, amphipathicity) (Komin et al., 2017). For example, melittin, a MAP present in bee venom, induced reversible BBBO following intra-arterial injection in a mouse model (Linville et al., 2021).

### 6.2. Alcohol

Alcohol is completely miscible with water and has a moderate lipophilicity (logP_oct_ ≈ −0.1) resulting in rapid passive diffusion across the BBB. Based on uptake studies in red blood cells, the permeability of ethanol is estimated to be around 10^−3^ cm s^−1^ (Brahm, 1982), much higher than most other small molecule drugs (Summerfield et al., 2007).

While early reports were inconclusive [summarized in Pan et al. (2008)], recent studies have found that chronic alcohol consumption causes BBB breakdown. Post-mortem brain tissue from individuals with alcoholism displays loss of basement membrane and tight junctions, increased MMP activity, and immune cell infiltration (Rubio-Araiz et al., 2017). In animal models of chronic alcohol consumption, leakage of blood components into the brain is observed, which may exacerbate prior neurodegeneration (Rubio-Araiz et al., 2017; Wei et al., 2019). *In vitro* studies suggest that ethanol-induced BBB dysfunction is mediated by oxidative damage to BMECs, causing loss of basement membrane, disrupted tight junctions, and increased immune cell infiltration (Haorah et al., 2005, 2008). Studies in mouse models of fetal alcohol spectrum disorders (FASD) suggest alcohol exposure can result in BBB dysfunction (Popova et al., 2023). Two photon imaging in the parietal lobe of 7–8 week male offspring revealed BBB leakage of 10 kDa and 70 kDa dextran in the FASD model but not in healthy controls (Tufa et al., 2017). Alcohol consumption can increase cerebral blood flow, even in low doses (Stendel et al., 2006), which may increase risk and worsen prognosis following hemorrhagic stroke (Liew et al., 2016). A side effect of chronic alcohol consumption is hyperammonemia, which results in increased levels of ammonia in circulation. Since the pK_A_ for the NH_4_^+^/NH_3_ equilibrium is 9.24, the concentration of NH_3_ in blood is very low. However, NH_3_ can passively diffuse across the BBB, where the NH_4_^+^/NH_3_ equilibrium is reestablished, resulting in accumulation of NH_4_^+^ in the brain. NH_4_^+^ is toxic to neurons and blood concentrations greater than 60 μM can be clinically significant with increasing concentrations resulting in coma or death (Cohn and Roth, 2004).

### 6.3. Stimulants and opioids

Acute and chronic exposure to stimulants such as methamphetamines or cocaine can result in compromised BBB function (Kousik et al., 2012; Sajja et al., 2016). Methamphetamines induce hyperthermia, hypertension, brain edema, seizures, and BBB breakdown causing severe neurotoxicity (Bowyer and Ali, 2006; Bowyer et al., 2018). Extravasation of blood components into the brain parenchyma is attributed to severe oxidative damage to BMECs (Ramirez et al., 2009), which causes downregulation/loss of tight junctions (Ramirez et al., 2009; Martins et al., 2011) and MMP-9 mediated degradation of the basement membrane (Martins et al., 2011; Urrutia et al., 2013). This toxicity is strongly linked to brain/body temperature, with the BBB intact in the absence of hyperthermia (Bowyer and Ali, 2006). Upregulation of TNF-α, increased immune cell infiltration (which promotes HIV-1 neuroinvasion), downregulated glucose transporter activity, and altered efflux pump expression (Ramirez et al., 2009; Coelho-Santos et al., 2015; Sajja et al., 2016), and may further exacerbate BBB damage and neurotoxicity. Therapies which protect from BBB disruption include antioxidant treatment, TNF-α inhibition, and MMP inhibition (Ramirez et al., 2009; Martins et al., 2011; Urrutia et al., 2013; Coelho-Santos et al., 2015). Cocaine exposure results in BBB disruption through similar mechanisms (Kousik et al., 2012; Sajja et al., 2016). In a study of opioids, BBB dysfunction was associated with withdrawal rather than acute consumption (Zhu et al., 2023). In individuals with opioid use disorder, the withdrawal score was correlated with the concentration of fragile-like regulatory T cells (Tregs). In a mouse model, opioids caused BBB disruption via downregulation of astrocyte-derived fatty-acid-binding protein 7 (Fabp7), resulting in Treg transmigration into the nucleus accumbens (NAc) and ultimately promoting withdrawal symptoms.

### 6.4. Anesthesia

General anesthesia and surgery can induce cognitive impairments in animal models, with effects in humans still controversial (Vutskits and Xie, 2016). Multiple studies have linked anesthesia-induced cognitive impairments to BBB disruption (Tetrault et al., 2008; Yang et al., 2017; Cao et al., 2018). Interestingly, opening of the BBB from anesthesia exhibits hallmarks of osmotic disruption, including brain edema, leakage of blood components, and heterogeneity of opening across brain regions (Tetrault et al., 2008). In addition, due to the range of anesthetics and doses, different dynamics of BBBO are observed (Tetrault et al., 2008; Acharya et al., 2015; Yang et al., 2017; Cao et al., 2018). Isoflurane, a common inhaled anesthetic, is the most widely studied in relation to BBB damage and results in dose-dependent effects in animal models (Tetrault et al., 2008). Upregulation of inflammatory cytokines, angiogenic stimuli (VEGF and HIF-1α), and MMPs have been implicated in anesthesia-induced BBB disruption, along with downregulation of TJs which leads to increased paracellular permeability (Wu et al., 2012; Cao et al., 2015, 2018; Yang et al., 2017). Aged mice are particularly vulnerable to anesthesia-induced BBB disruption (Acharya et al., 2015; Yang et al., 2017), which has been linked to increased risk of neurodegeneration and dementia (Vutskits and Xie, 2016).

## 7. Endogenous factors

### 7.1. Microbiome and gut – brain axis

The gut – brain axis (GBA) enables bidirectional communication between the gastrointestinal tract and the brain either directly, via the vagus nerve, or indirectly, via soluble factors in circulation, which implicates the BBB (Parker et al., 2020; Tang et al., 2020). The gut microbiome transforms dietary components into a diverse range of molecules, including short chain fatty acids (SCFAs, e.g., butyrate, acetate, propionate), neurotransmitters, neuropeptides, and serotonin. Changes in the gut microbiome can result in dysfunction in pathways along the gut – brain axis, ultimately resulting in BBB dysfunction and neuroinflammation (secretion of TNF-α, INF-γ, IL-6). In a comparison of germ-free and pathogen-free mice, the permeability to Evans blue/albumin was higher in germ-free mice and was associated with a decrease in occludin and claudin-5 in BMECs (Braniste et al., 2014). Rescue studies found that colonization of germ-free mice with flora from pathogen-free mice resulted in decreased BBB permeability and upregulated the expression of tight junction proteins. Gavage with bacterial strains that produce butyrate, acetate, and propionate had a similar effect.

Changes in the gut microbiome can result in increased intestinal permeability (leaky gut syndrome), often due to factors such as diet, lifestyle, chronic stress, and antibiotic treatment. Leaky gut is associated with chronic systemic inflammation, and in the hygiene hypothesis, is one of several factors that contribute to chronic inflammatory diseases (Fasano, 2020).

### 7.2. Diet

With expanding knowledge of the gut-brain axis, diet is emerging as a key endogenous factor that influences brain and cerebrovascular health. Body mass index and obesity are correlated with risk of BBB disruption in humans (Gustafson et al., 2007). Several studies have found increased BBB permeability in the hippocampus in animal models in response to high fat or high cholesterol diets (Chen et al., 2008; Kanoski et al., 2010; de Aquino et al., 2018).

A wide array of dietary supplements have been investigated for their promotion of BBB health. Vitamin D, vitamin B (1, 12, 5, 9), Mg, and omega 3 fatty acids are thought to be neuroprotective and may directly or indirectly promote BBB health (Roy et al., 2022). Antioxidant or anti-inflammatory molecules, such as curcumin, resveratrol, sulforaphane, may have a similar effect. In patients with mild cognitive impairment, orally administered vitamin B12/B6 reduced the ratio of tau protein to albumin in CSF, implying improved barrier function (Lehmann et al., 2003). In an *in vitro* BBB model with a monolayer of primary BMECs in astrocyte conditioned media, Mg increased TEER and reduced permeability, and increased the transcytosis of amyloid beta (Aβ) (Zhu et al., 2018). In an *in vitro* multiple sclerosis (MS) model, vitamin D reduced the effects of TNF-α on BMEC barrier function (Takahashi et al., 2017). Studies in mice and humans have shown that omega-3 fatty acids reduce BBB disruption associated with aging and surgery-induced impairment (Barnes et al., 2021; Yang T. et al., 2022).

### 7.3. Aging

Aging is associated with accumulation of cell damage, including oxidative stress, epigenetic changes, dysregulation of cell signaling and inflammatory responses, and senescence. While healthy aging is not well defined, it can be considered as age-related changes that result in minimal decline in cognitive function (Menassa et al., 2023). MRI imaging studies suggest small but measurable increases in BBB permeability in healthy aging, although the magnitude varies considerably between individuals (Montagne et al., 2015). Transporters such as Glut-1, LAT-1, P-gp, and LRP-1 are also downregulated in aging. LRP-1 is important for brain-to-blood transport of Aβ peptide and hence its downregulation can lead to subsequent Aβ accumulation (Jaeger et al., 2009). Aβ is also a P-gp substrate, and hence downregulation of this efflux pump can also contribute to accumulation (Hartz et al., 2010). The downregulation of P-gp is also thought to increase sensitivity to CNS-active molecules that are efflux substrates (Banks et al., 2021). Transcriptomic studies in mice have found age related increases in the fraction of BMECs that express senescence-related genes (Kiss et al., 2020), and downregulation of the senescence regulator sirtuin-1 is associated with increased BBB permeability (Stamatovic et al., 2019).

Studies in animal models and post-mortem human tissue have shown a wide array of structural and functional changes during healthy aging. Changes include a decrease in microvascular density (~10–30% in the prefrontal cortex and hippocampus), a decrease in brain arteriole density (~40% in the cerebral cortex), thickening of the basement membrane, loss of SMCs, pericyte degeneration/loss, swelling of astrocytic end-feet, defects in tight junctions, increased vesicular transport, and increased permeability (Sonntag et al., 2007; Brown and Thore, 2011; Murugesan et al., 2012; Montagne et al., 2015; Erdo et al., 2017; Goodall et al., 2018; Reagan et al., 2018; Yang et al., 2020). Based on these observations, it is apparent that some level of BBB dysfunction can be tolerated with minimal changes in cognitive function. However, co-occurring pathologies, such as inflammation, can result in more advanced pathological symptoms. This has been demonstrated in mouse models, where BBB function is diminished in healthy aged mice, but cognitive decline is only apparent after an inflammation challenge (Wen et al., 2020). This is analogous to the “two-hit” vascular hypothesis for Alzheimer’s disease.

Aging is associated with changes in protein composition in circulation. Several studies have shown that infusion of plasma from young mice improves cognitive function in aged mice (Wyss-Coray, 2016). Transcriptomic studies in mice have shown significant differences in aged mice compared to young mice, with upregulation of pathways associated with innate immunity and oxidative stress response (Chen M. B. et al., 2020). These effects were most pronounced in capillaries compared to arterioles and venules. These changes were replicated in young mice 4 days after infusion of plasma from aged mice. Furthermore, infusion of plasma from young mice into aged mice resulted in rejuvenation on the transcriptome. In particular, Mfsd2a was downregulated in BMECs in aged mice, resulting in increased protein levels of caveolin-1 and implying increased non-specific vesicular transport.

### 7.4. Social stress

Studies in a male mouse model of social stress showed ultrastructural defects and decreased levels of claudin-5 in BMECs in capillaries in the nucleus accumbens compared to unstressed mice (Menard et al., 2017). The nucleus accumbens plays an important role in stress responses and mood disorders. In MRI imaging, increased levels of Gd contrast agent were observed in the nucleus accumbens and hippocampus of stressed mice. Treatment for 35 days with the antidepressant imipramine rescued these changes in the BBB. Conditional knockdown of CLDN5 in the nucleus accumbens did not induce depression behaviors but did induce depression following microdefeat at levels below the threshold to induce depression compared to mice without CLDN5 knockdown. Increased levels of IL-6 were observed in blood and the nucleus accumbens (but not hippocampus) of stressed mice. Reduced *CLDN5* expression was also observed in post-mortem tissue of unmedicated individuals with major depressive disorder compared to healthy controls. These results provide evidence that social stress increases paracellular permeability in the nucleus accumbens resulting in accumulation of inflammatory cytokines (e.g., IL-6) and the development of depression-like behaviors (Menard et al., 2017). Similar experiments showed leaky BBB (10 kDa dextran) in the prefrontal cortex (but not nucleus accumbens) of stressed female mice (Dion-Albert et al., 2022). The pre-frontal cortex is responsible for executive function and decision making. Decreased claudin-5 was observed in microvessels of stressed mice compared to controls. Loss of claudin-5 was also observed in the pre-frontal cortex of post-mortem tissue from females who died by suicide compared to nonpsychiatric controls (Dion-Albert et al., 2022). In a panel of biomarkers for vascular health, soluble E-selectin was increased in stressed mice.

### 7.5. Glymphatic clearance

Fluid and solute exchange across the BBB is highly restricted and hence waste products and debris produced in the brain are: (1) transported across BMECs into circulation by passive diffusion (e.g., CO_2_), (2) effluxed across BMECs into circulation (e.g., soluble Ab, tau), (3) recycled by cells in the brain (e.g., microglia), or (4) transported out of the brain into the lymphatic system. In the glymphatic system model, removal of waste products into the lymphatic system occurs via fluid flow from the CSF along the perivascular spaces (PVS) around penetrating arteries (mediated by aquaporin-4 (AQP4) water channels in astrocyte end-feet), transport via interstitial flow (e.g., white matter tracts), efflux along the perivascular space around venules, and finally drainage into meningeal lymphatic vessels (Bohr et al., 2022). Two-photon microscopy studies with tracers have identified a fluid filled perivascular space around penetrating arterioles between the smooth muscle cells and astrocyte end-feet (Mestre et al., 2020). In post-mortem imaging the PVS collapses and hence the region between SMCs and astrocyte end-feet is only occupied by basement membrane. Whether there is a continuous PVS along the arterio-venous axis enabling a direct route for transport remains to be firmly established (Hannocks et al., 2018; Ferris, 2021).

While sleep and exercise promote efficient transport, perturbations that reduce the efficiency of the glymphatic system have the potential to induce BBB dysfunction. For example, reduction of the perivascular space can reduce glymphatic flow and hence result in accumulation of waste products or toxins (Bohr et al., 2022). It has been speculated that conditions such as hypertension and inflammation can impact the PVS and hence impair glymphatic clearance. For example, MRI imaging has shown that small vessel disease (SVD) is associated with microbleeds and enlarged PVS around arterioles which is thought to be due to downstream accumulation of waste products reducing flow (Brown et al., 2018). SVD is associated with accumulation of advanced glycation end products (AGEs) and activation of the corresponding receptor (RAGE). Accumulation of RAGE is associated with hypertension, aging, inflammation, and oxidative stress (Brown et al., 2018). Similarly, Since the glymphatic system plays a role, along with efflux pumps, in the removal of soluble Aβ, disruption of glymphatic clearance can lead to accumulation of Aβ plaques in the basement membrane.

## 8. Biochemical factors

### 8.1. Oxidative stress

Oxidative stress arises from an excess production of free radicals such as reactive oxygen species (ROS) and reactive nitrogen species (RNS). Free radicals are intermediates in cellular energy production and function as second messengers in various signaling pathways (Forman et al., 2004). Accumulation of free radicals, for example under hypoxic conditions, is counterbalanced by endogenous anti-oxidant mechanisms. However, under pathological conditions free radical production can overwhelm the antioxidant capacity resulting in accumulation of oxidized proteins, lipids, and nucleic acids. Free radicals and cellular oxidative stress can be triggered by an array of physical and biological processes including changes in metabolism with cell age, inflammation and immune responses, environmental factors such as smoking and air pollution, and exposure to UV or ionizing radiation (Golden et al., 2002; Auten and Davis, 2009; Al-Gubory, 2014; Carraro et al., 2018). For example, ROS and RNS are produced by phagocytic cells in response to infection. Secreted ROS and RNS can induce oxidative stress and tissue damage if not consumed by antioxidant mechanisms. The brain is particularly sensitive to oxidative stress due to its high metabolic demand, the involvement of ROS and other free radicals in some neuronal signaling pathways, ROS generation by activated microglia and astrocytes, and reduced antioxidant capacity compared to other tissues (Cobley et al., 2018). BMECs are susceptible to oxidative stress due to their high mitochondrial density (Cobley et al., 2018).

Oxidative stress is a key component in the etiology of many neurodegenerative pathologies, including Alzheimer’s disease (AD), MS, stroke, and TBI (Rodriguez-Rodriguez et al., 2014; Rao et al., 2018). Several mechanisms can promote downregulation of TJs, including ROS-induced cleavage of cysteine residues in claudin-5 and occludin, and the degradation of basement membrane through downstream production of MMPs (Rempe et al., 2016; van Leeuwen et al., 2020). These and other studies suggest a correlation between oxidative stress, loss of barrier function, and downstream neurodegeneration and disease. Since oxidative stress is a balance between production and antioxidant capacity, the impact on BBB dysfunction involves common intrinsic mechanisms including inflammation and angiogenesis. These pathways are activated across many diverse perturbations. As a result of the role of ROS in normal cell signaling, the impact of free radical production is highly dose-dependent. At low concentrations (< 1 μM), hydrogen peroxide (H_2_O_2_), a common ROS, promotes angiogenic activity in brain microvascular endothelial cells, while higher doses (> 100 μM) result in increased permeability of cell monolayers. Very high doses (> 10 mM) result in apoptosis (Anasooya Shaji et al., 2019; Chung et al., 2022).

While the generation of excess ROS in the brain represents a pathological perturbation on the BBB, various strategies have been explored for improving antioxidant capacity, particularly in stroke. Antioxidant therapies include inhibition of ROS producing enzymes, free radical scavengers (Tirilazad), increasing concentrations of endogenous free radical antioxidants (e.g., superoxide dismutase), and antioxidant supplementation (e.g., vitamin C, vitamin E, N-acetylcysteine (NAC), etc.).

### 8.2. Inflammation

An inflammatory response by the innate immune system involves interplay between the initial insult (infection or injury), the local tissue microenvironment, and the peripheral immune system. A successful inflammatory response results in elimination of pathogens and promotes tissue remodeling and repair. Neuroinflammation can be caused by a wide range of perturbations including pathogens, injury (e.g., TBI, stroke), endogenous factors (e.g., mutations or protein aggregation), autoimmune disease, systemic chronic inflammation, mental stress, metabolic disorders (e.g., diabetes, obesity), lifestyle factors (e.g., gut-brain axis), and environmental factors (Figure 8) (Sun et al., 2022). Sustained neuroinflammation can inhibit repair and ultimately lead to neurodegeneration and cognitive impairment.

The healthy BBB limits interactions between cells of the peripheral immune system and the brain. Evidence suggests that the machinery of immune cell recruitment and transmigration in BMECs specifically allows a subset of activated T cells in circulation to migrate across the healthy BBB (in the absence of inflammation) into the perivascular space (PVS) surrounding post-capillary venules or into the subarachnoid space (Engelhardt et al., 2017; Mapunda et al., 2022). Post-capillary venules sustain a low wall shear stress increasing the probability of capture and adhesion. Current models suggest that reactivation of these T cells following interactions with antigen presenting cells in the PVS promotes migration across the glia-limitans into the brain parenchyma (Mapunda et al., 2022; Aydin et al., 2023). Microglia, the primary resident immune cells in the brain, along with astrocytes, are responsible for many functions in the brain including the response to infection or injury.

There are four general causes of inflammation in the brain that can lead to BBB dysfunction: (1) neuroinflammation initiated in the brain parenchyma, which subsequently causes BBB dysfunction (e.g., MS), (2) neuroinflammation induced by systemic inflammation (e.g., sepsis), and (3) neuroinflammation caused by injury to the brain that results in simultaneous BBB disruption and neuroinflammation (e.g., TBI), and (4) neuroinflammation initiated by systemic inflammation and brain pathologies (e.g., Ab plaques, tau tangles) that together drive BBB dysfunction. These causes of inflammation can occur simultaneously, for example, familial mutations associated with neurodegenerative diseases (NDDs) result in production of toxic proteins both peripherally and in the brain. A current challenge is in identifying conditions or thresholds that prevent the resolution of the inflammatory response and lead to neurodegeneration, and how these thresholds may be impacted with other endogenous or exogenous perturbations such as oxidative stress, age, or lifestyle factors.

Brain hypoperfusion and ischemia lead to a cascade of events including generation of ROS, downregulation of TJs, loss of contact of astrocytic end-feet, loss of basement membrane, activation of astrocytes and microglia, and release of inflammatory cytokines, which together can result in entry of blood components into the brain. Reperfusion enables extravasation of leukocytes in circulation, further increasing release of inflammatory cytokines. Therefore, in MS, TBI, and stroke, the primary injury is followed by inflammation which leads to secondary neurodegeneration. In other diseases, including Alzheimer’s disease, amyotrophic lateral sclerosis, Parkinson’s and Huntington’s disease (HD), inflammation amplifies the pathological response and may play an important role in initiation.

The influence of systemic inflammation on BBB function is dependent on the concentration of pro-and anti-inflammatory factors, the identity of immune cells in circulation, and the duration of inflammation. At a sufficiently high level, inflammation can induce loss of barrier function, increased paracellular permeability, increased lymphocyte trafficking, and immune cell infiltration. For example, several studies in mouse models have shown a threshold concentration of LPS of around 3 mg/kg is required to observe increased solute permeability into the brain (Banks et al., 2015). Studies of Lucifer yellow permeability in rat pial capillaries (13–19 μm diameter) showed a 10-fold increase in permeability from 2 × 10^−7^ to 2.4 × 10^−6^ cm s^−1^ during perfusion via the carotid artery with 10 μM histamine (Easton et al., 1997). In addition, retrospective human studies have found that patients with an “abnormally inflammatory” CSF—as indicated by lymphocyte count, C-reactive protein, and erythrocyte sedimentation rate, among others—consistently had high albumin accumulation in the brain, indicating measurable disruption of the BBB (Elwood et al., 2017). Assessment of exposure to systemic inflammation is complicated by the fact that several cytokines including IL-1, IL-6, TNF-α, INF-γ, along with growth factors BDNF, EGF, and FGF are transported across the healthy BBB into the brain (Banks et al., 2009). Growth factors can also contribute to BBB leakage by promoting angiogenesis (Argaw et al., 2009, 2012).

Sepsis, an extreme reaction to infection, can lead to neuroinflammation and sepsis-associated encephalopathy (SAE), which is a common neurological complication. In animal models of exacerbated immune response using cecal ligation and punction or IP injection of LPS (10 mg/kg), increased BBB permeability (Evans blue, Gd contrast agent, etc.) is observed 12–24 h following the procedure (Gao and Hernandes, 2021). For example, in a cecal ligation and punction model in rats, immune cell adhesion in the cerebrovasculature was observed 6 h following induction (Comim et al., 2011; Barichello et al., 2021). Increased levels of MMP-2 and MMP-9 in the hippocampus and cortex at 12 h preceded BBB breakdown and increased levels of IL-1β, TNF-α, and IL-6. Persistent activation of microglia and release of ROS and RNS further increased cytotoxicity. In humans, brain tissue samples obtained from deceased sepsis patients revealed a significant downregulation of the TJ proteins occludin, claudin-5, and zonula occludens-1 (ZO-1) in microvascular endothelial cells (9) (Erikson et al., 2020), suggesting impaired BBB function.

While biomarkers such as TNF-α, IL-1β, IL-6, and CRP are associated with acute inflammation there are no recognized biomarkers for chronic inflammation (Furman et al., 2019). Systemic chronic inflammation (SCI) can be caused by various environmental and lifestyle factors and can lead to a wide range of diseases including hypertension, cardiovascular disease, and neurodegenerative disorders (Furman et al., 2019). In support of the link between SCI and neurodegenerative disease, individuals with rheumatoid arthritis treated with a TNF-α inhibitor had significantly lower incidence of AD (Chou et al., 2016). Furthermore, SCI increases with aging and is thought to be caused by cellular senescence and the development of a senescence-associated secretory phenotype (SASP) (Furman et al., 2019). Risk factors for SASP include intrinsic factors (DNA damage, oxidative stress) and extrinsic environmental and lifestyle factors (diet, gut microbiome, chronic infections, exposure to toxins, etc.).

### 8.3. Angiogenesis

**Figure 9:**
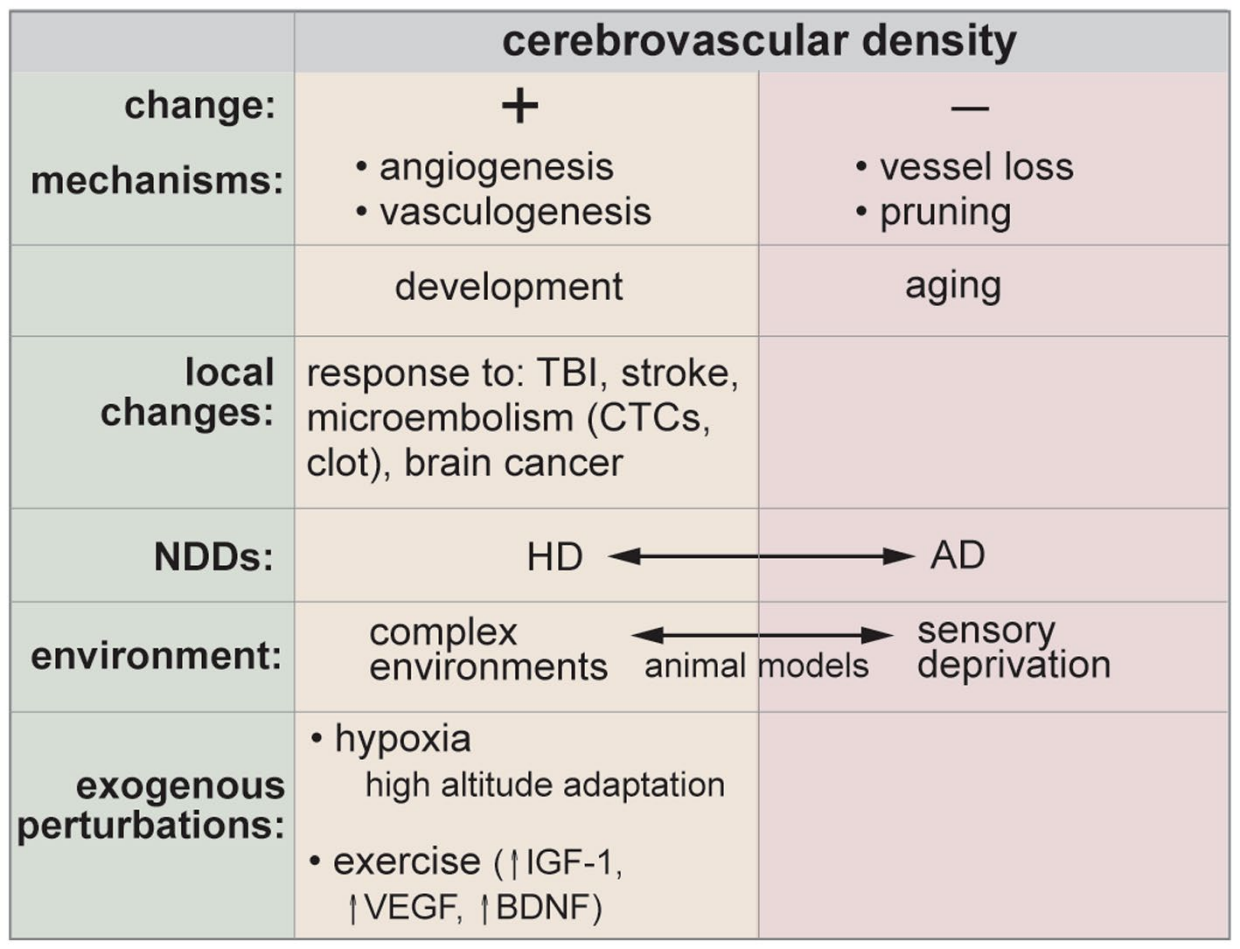
In the adult brain there is little change in cerebrovascular density, however, various injuries, pathological conditions, or environmental factors can promote angiogenesis or vessel loss and pruning. CTCs, circulating tumor cells. NDDs, neurodegenerative diseases. HD, Huntington’s disease. AD, Alzheimer’s disease.

The vasculature in the adult brain is largely quiescent, however, angiogenesis is associated with stroke (Croll and Wiegand, 2001; Brown et al., 2007; Jiang et al., 2018), brain cancer (Jain et al., 2007), TBI (Shlosberg et al., 2010; Salehi et al., 2017), exercise (Swain et al., 2003; Pereira et al., 2007), and high altitude adaptation (Figure 9; Park et al., 2016). In individuals with Huntington’s disease (HD), microvascular density is increased up to two-fold in the putamen compared to age-matched healthy controls, implying disease-associated angiogenesis (Drouin-Ouellet et al., 2015). In contrast, a loss in vascular density is associated with AD implying vessel loss and vascular pruning, although little is known about the impact of these processes on BBB function. Despite its importance in response to these perturbations, very little is known about the mechanisms of angiogenesis in the mature brain. A better understanding of the mechanistic details of angiogenesis in the mature brain is generally thought to be key to developing improved therapies for vascular repair.

In the classical model of sprouting angiogenesis in capillaries or venules in the brain, the local production of angiogenic factors activates BMECs, resulting in degradation of basement membrane and detachment of pericytes and other supporting cells (Conway et al., 2001; Carmeliet and Jain, 2011). Endothelial tip cells guide sprout growth toward another vessel, ultimately resulting in anastomosis and perfusion. Maturation of the sprout involves recruitment of pericytes (or other mural cells) and generation of new basement membrane. While the classical model depicts pericytes as passive bystanders, some studies have shown that pericytes can play an active role in angiogenesis, leading sprout growth and promoting the migration of stalk cells (Coelho-Santos et al., 2021; Zhao et al., 2023).

A wide range of growth factors are thought to regulate angiogenesis in the brain, including VEGF-A, bFGF, platelet derived growth factor (PDGF), insulin-like growth factor, Wnt, brain-derived neurotrophic factor, among others. The most widely studied are VEGF, bFGF, and PDGF, which are expressed by many diverse cell types. VEGF and bFGF promote angiogenesis in a wide range of tissues, including brain (Eelen et al., 2020; Trembath et al., 2020). PDGF-BB, secreted mainly by endothelial cells, plays an important role in recruiting pericytes to newly formed blood vessels and maintaining BBB integrity (Zhao et al., 2015). In stroke, hypoxia is thought to be the master regulator of angiogenesis with low O_2_ levels upregulating hypoxia inducible factors (HIFs), which in turn upregulates VEGF.

Many growth factors involved in angiogenesis have been shown to affect barrier function. VEGF downregulates TJs leading to increased BBB permeability (Argaw et al., 2012). In a mouse model, injection of 3 μL of 20 ng μL^−1^ VEGF-A into the brain resulted in decreased claudin-5 and occludin expression in BMECs and increased entry of albumin 24 h after injection, although no differences were detectable after 7 days, indicating that the disruption was reversible (Argaw et al., 2009).

Pathways associated with angiogenesis remain the targets for the development of pro-and anti-angiogenic therapies (Carmeliet and Jain, 2011; Durand et al., 2017; Eelen et al., 2020). Therapeutic angiogenesis, largely based on the prototypical growth factor VEGF, has been widely explored for treatment of non-brain ischemia and ischemic stroke (Semenza, 2006; Moccia et al., 2020). However, a consequence of therapeutic angiogenesis in the brain is increased paracellular permeability during vascular remodeling.

## 9. Pathogens

A broad range of pathogens including bacteria, fungi, and viruses can enter the brain leading to BBB dysfunction and increased risk of CNS infection and neuroinflammation. Despite the fact that these pathogens can enter the brain, the BBB remains a limitation in treating the infection.

Some of the most common bacterial species that cause CNS infection include *Escherichia coli, Streptococcus pneumoniae, Haemophilus influenzae*, and *Neisseria meningitidis* (Kim, 2006). Infection by these pathogens lead to bacterial meningitis which is inflammation of the protective membrane surrounding the brain and spinal cord. Although there is an incomplete understanding of how bacteria penetrate the BBB to cause meningitis, several factors are linked to successful invasion, including high levels of bacteria in circulation, adhesion to BMECs, and translocation across the BBB (Moxon and Ostrow, 1977; Bell et al., 1985; Kim, 2001, 2002, 2003). Currently, the main therapeutic strategies for treating bacterial meningitis include administering antibiotics as well as reducing inflammatory response using adjunctive drugs such as dexamethasone (De Gans and Van de Beek, 2002; Van de Beek et al., 2012).

Another example of bacterial infection in the CNS is Lyme neuroborreliosis, which is caused by the spirochete *Borrelia burgdorferi* (*Bb*) (Rupprecht et al., 2008; Kullberg et al., 2020). Lyme neuroborreliosis is reported in up to 12% of patients with Lyme disease, a tick-borne disease that is prevalent throughout North America, Europe, and Asia (Koedel et al., 2015). Common symptoms of Lyme neuroborreliosis include headaches, cranial nerve palsy, numbness and tingling, and fatigue (Pachner and Steiner, 2007; Schwenkenbecher et al., 2017). The exact method in which *Bb* are able to cross the BBB remains unclear (Comstock and Thomas, 1989; Szczepanski et al., 1990). However, in contrast with *E. coli* and *S. pneumoniae*, the concentration of *Bb* in human blood during infection is very low (≤ 0.1 per mL), indicating that the mechanism of infection is not consistent across bacterial species (Wormser et al., 2001; Pritt et al., 2016).

The incidence of fungal infections has increased as a result of an increase in the use of immunosuppressive drugs, autoimmune diseases, and invasive medical interventions (Lionakis and Levitz, 2018). Fungi can enter the brain either by direct inoculation (TBI, surgical intervention) or by crossing the BBB from circulation. Receptors for fungi (e.g., TLR, CLR) on astrocytes and microglia can initiate clearance or recruit immune cells via expression of cytokines (Snarr et al., 2020). However, if the infection is not cleared, neuroinflammation can lead to encephalitis and neuronal damage. Prevalent fungal species known to cause CNS infections include *Cryptococcus neoformans, Candida albicans, Aspergillus fumigatus*, and *Zycomycetes* (Lionakis and Levitz, 2018; Snarr et al., 2020). The WHO fungal priority list identifies *Cryptococcus neoformans, Candida albicans*, and *Aspergillus fumigatus* as “critical” for R&D and public health action (WHO fungal priority pathogens list to guide research, 2022). While *Candida* and *Aspergillus* are common fungal pathogens, CNS infections are relatively rare. However, for individuals with CNS infection the prognosis is poor. *Cryptococcus* infection in the brain has a very high mortality rate if untreated. While relatively little is known about the exact mechanisms of transport across the BBB, evidence suggests that *Cryptococcus* can enter the brain via infected monocytes or T cells in post-capillary venules, or by paracellular transport in capillaries (Snarr et al., 2020).

Common viruses that are known to infect the brain and disrupt the BBB include human immunodeficiency virus-1 (HIV-1), West Nile virus (WNV), herpes simplex virus-1 (HSV-1), and rabies virus (Spindler and Hsu, 2012). In the brain, the virus can replicate in various cell types leading to infection unless it is cleared by an immune response. Viral infection can lead to encephalitis and other pathological conditions such as meningitis (Klein et al., 2019). There are a variety of mechanisms by which viruses can enter the brain. The main route of entry for HIV-1 and WNV is via infected monocytes or T cells resulting in replication and infection of microglia (Wahl and Al-Harthi, 2023). As described previously, T cell migration into the perivascular space surrounding post-capillary venules is part of the limited immune surveillance in the brain. HIV invades the brain within ~2 weeks following infection (Wahl and Al-Harthi, 2023), where replication in the brain can lead to neuroinflammation and HIV-Associated Neurocognitive Disorder (HAND). HSV-1 and rabies, on the other hand, are thought to invade the brain through retrograde axonal transport (Schnell et al., 2010; Spindler and Hsu, 2012). In each of these cases, there is an increase in BBB permeability as well as inflammation (Toneatto et al., 1999; Wang et al., 2004; Phares et al., 2006). While some viruses, such as SV40, EV1, and cholera toxin B can cross endothelial barriers via caveola-mediated transcytosis, the suppression of this mode of transport in the healthy brain likely limits neuroinvasion (Klein et al., 2019). Several recent studies have found a correlation between exposure to viral infections (e.g., Epstein–Barr, viral encephalitis, influenza/pneumonia) and subsequent risk for neurodegenerative disease (Bjornevik et al., 2022; Levine et al., 2023).

## 10. Conclusion

BBB dysfunction is usually considered in the context of disease. Here we consider BBB dysfunction in the context of eight perturbations associated with BBB and brain health: mechanical forces, temperature, electromagnetic radiation, hypoxia, endogenous factors, exogenous factors, chemical factors, and pathogens. These perturbations may be endogenous or exogenous, pathological or therapeutic, or intended or unintended. The resultant outcome on BBB function depends on the dose (or magnitude) and duration of the perturbation. Understanding the influence of these perturbations is key to understanding the mechanisms and pathologies associated with BBB dysfunction and in developing new therapeutic strategies to treat diseases of the brain.

## Author contributions

NZ: Writing – original draft, Writing – review & editing. TC: Writing – original draft, Writing – review & editing. ZG: Writing – original draft, Writing – review & editing. JJ: Writing – original draft, Writing – review & editing. LL: Writing – original draft, Writing – review & editing. RL: Writing – original draft, Writing – review & editing. AP: Writing – original draft, Writing – review & editing. LW: Writing – original draft, Writing – review & editing. PS: Conceptualization, Funding acquisition, Writing – original draft, Writing – review & editing.
