# Corrigendum: The influence of physiological and pathological perturbations on blood-brain barrier function

**DOI:** 10.3389/fnins.2023.1328902

**Published:** 2024-03-21

**Authors:** Nan Zhao, Tracy D. Chung, Zhaobin Guo, John J. Jamieson, Lily Liang, Raleigh M. Linville, Alex F. Pessell, Linus Wang, Peter C. Searson

**Affiliations:** ^1^Institute for Nanobiotechnology, Johns Hopkins University, Baltimore, MD, United States; ^2^Department of Biomedical Engineering, Johns Hopkins University, Baltimore, MD, United States; ^3^Department of Chemical and Biomolecular Engineering, Johns Hopkins University, Baltimore, MD, United States; ^4^Department of Materials Science and Engineering, Johns Hopkins University, Baltimore, MD, United States

**Keywords:** blood-brain barrier, perturbations, dysfunction, brain health, neurovascular unit, brain pathologies

In the published article, there was an error in Table 1 as published. In row 6, column 3, “Ab” should be “Aβ”. In row 5, column 3, “Ab” should be “Aβ”. The corrected Table 1 and its caption Blood-brain barrier (BBB) components and examples of dysfunction appear below.

**Table 1:** Blood-brain barrier (BBB) components and examples of dysfunction.

**BBB function**	**Molecular constituents**	**Dysfunction**	**Examples**
Paracellular transport	Tight junctions (TJs) expressed by BMECs (e.g. claudin-5, occludin, ZO-1, …)	• ↓ TJs – ↑ paracellular transport • cell loss – ↑ paracellular transport • mechanical disruption - ↑ paracellular transport	Leaky brain:microbleeds or microhemorrhages (AD, CAA, stroke, TBI, healthy aging)
Passive transport	Small (< 500 Da) lipophilic molecules	see ATP-binding cassette efflux pumps (ABCs)	
Carrier mediated transport: solute carrier transporters (SLCs)	Energy transport (e.g. GLUT-1), amino acid transport (e.g. LAT-1), organic anion/cation transport (e.g. OATP1A2), nucleotides	↓ GLUT-1 - ↓ nutrient transport ↓ LAT-1 - ↓ protein & nucleotide synthesis, metabolism	Changes in respiration of cells in NVU
ATP-binding cassette efflux pumps (ABCs)	P-glycoprotein (P-gp, ABCB1), BCRP (ABCG2), MRP1 (ABCC1)	↓ P-gp - ↑ passive transport of substrates, ↓ clearance of Aβ	
Vesicular trafficking I receptor-mediated transport	Transferrin receptor (TfR), insulin receptor (IR), leptin receptor (LEP-R), low density lipoprotein receptor 1&2 (LRP1/2), receptor for advanced glycation end products (RAGE)	↓ LRP1 - ↓ clearance of Aβ and APOE 2/3	
Vesicular trafficking II adsorption mediated transport	Histone, albumin	↓ MFSD2A - ↑ caveola-mediated vesicular transport	Shift to non-specific transport in aging
Ion transporters	Sodium pumps, calcium transporters, and potassium channels	changes in ionic homeostasis	
Other processes involving BMECs	Wound healing response, activation	activation - ↑ adhesion molecules (e.g. ICAM-1)	
Supporting cells	Loss or degeneration of SMCs, PCs; detachment of astrocytic end-feet	↓ signaling between BMECs and supporting cells	AD
Basement membrane	Endothelial, parenchymal (collagen IV, laminin, perlecan, agrin, nidogen)	↑ thickness in aging	Aging, AD

There was also an error in Section 3. Temperature, paragraph 1, “when CBF is lowest” should be “when CBF is highest”. The corrected paragraph appears below:

The average core body temperature (Tc) of healthy individuals is around 36°C (Mackowiak et al., 1992; Mackowiak and Worden, 1994; Sund-Levander and Grodzinsky, 2009; Protsiv et al., 2020). The average temperature of the brain is typically 1–2°C higher than Tc due to its high metabolic rate (Bain et al., 2015). Recent studies suggest that brain temperature varies with brain region and age, with temperatures as high as 40°C measured in the thalamus of healthy adults (Rzechorzek et al., 2022). The average brain temperature shows diurnal cycles, with the lowest temperature at night when CBF is highest, although these cycles are compromised with aging (Rzechorzek et al., 2022).

The authors apologize for these errors and state that this does not change the scientific conclusions of the article in any way. The original article has been updated.

